# Personal online social networks as moderators of the association between loneliness and quality of life in Polish adults aged 50+

**DOI:** 10.1038/s41598-025-24545-z

**Published:** 2025-11-19

**Authors:** Barbara Woźniak, Michalina Gajdzica, Paulina Sekuła, Karolina Majdak, Beata Tobiasz-Adamczyk, Katarzyna Zawisza

**Affiliations:** 1 Jagiellonian University Medical College, Chair of Epidemiology and Preventive Medicine, Department of Medical Sociology, Krakow, Poland; 2 Jagiellonian University Medical College, Chair of Epidemiology and Preventive Medicine, Department of Epidemiology, Krakow, Poland

**Keywords:** Online social networks, Loneliness, Quality of life, Middle-aged adults, Older adults, Moderation analysis, Online community, Epidemiology, Public health, Quality of life

## Abstract

In response to a critical public health issue related to the need of better understanding the underlying consequences of social isolation and loneliness this study investigates the moderating role of personal online social networks in the association between loneliness and QoL in Polish men and women aged 50 years or older. The cross-sectional study COURAGE-CAD was done in 2024. The analysis included 1,802 face-to-face interviews conducted with randomly selected people (50+) from among general Polish population. Multiple linear regression models with interaction terms were used. Among women who were members of online community the association between loneliness and QoL was not statistically significant contrary to women who did not use the internet or use it but without being a member of online community. All models controlled for age and further for sociodemographic variables, health, functional status gave similar results. Additional adjustment for the level of social networks (offline) also did not change the results. The study showed that encouraging middle-aged and older adult women to join online communities might mitigate the negative effect of loneliness on their QoL.

**Supplementary Information:**

The online version contains supplementary material available at 10.1038/s41598-025-24545-z.

## Introduction

The concept of social networks was introduced in the 1950s by Barnes and Bott for the purpose of analysis of social ties that cut across traditional categories of kinship, neighborhood and class groups; this concept provided a way to view the structural properties of social relationships^1^. Traditionally, social networks are understood as the quantifiable relationships between individuals, families, groups, etc., that are held together by a common need, goal, or interest, and that facilitate social engagement and access to social support^2–4^. In social science both egocentric networks (i.e. network surrounding an individual) as well as the connections between various social networks (i.e. networks of networks) are analyzed^2^. The latter, i.e. sociocentric approach, involves the analysis of the relationships between people in a given community/society. The egocentric approach is focused on personal networks and is commonly used in research on social determinants of health and well-being.

Social networks analysis focuses on the patterns of relationships between individuals, positions or groups; the network itself is a structure that consists of objects and connections between them. Such analysis requires determining the nature of those objects and the characteristics of the connecting ties^5^ as well as identification of the flows of resources that are exchanged within the network. These flows exchanged between individuals can be of different nature, including flows of emotions (e.g. sympathy, approval, respect), symbols (such as information, ideas, values, norms) and goods (e.g. money and other material goods and services); even the connections themselves are recognized as resources^2,6^. Social networks measures cover various aspects of network characteristics, including quantitative dimensions (e.g. network size, density, structure, contacts frequency), qualitative dimensions (e.g. emotional bond, social support, support satisfaction) and alter members (e.g. family, friends, partner, colleagues, neighbors)^3^.

In the last three decades new challenges in the studies on social networks have emerged. Contemporary societies, that are referred to as information societies, are characterized by increased activity of social actors in the digital world^7^. Information and communication technologies (ICT), penetrating virtually every aspect of social life, generate new forms of community formations^8^, including online social networks, like those established through the social networking sites (SNS), also known as “social media”. These are specific tools developed to enable creation of new and sustain already existing social relationships and to communicate within social networks^9^. SNSs have been popular throughout the world since the early 2000s (and since 2007, its’ popularity has skyrocketed due to common usage of smart mobile devices and widespread internet access;^10,11^, firstly among younger generations, but nowadays the use of SNSs is significantly increasing also among older adults^12^. For instance, according to the analyses of the population of Polish social media users (data for 2024), more than 6,6 million of the real users of the social media sites was recorded in the age group of 55–75. This age group devotes more than 34% of their online time to the SNSs. Among the SNSs that are the most popular in the Polish older adults there are Facebook (6,2 million users) and YouTube (5,8 million users); women are using these platforms more often than men^13^. Data from the systematic review of the papers about the SNSs and the experience of older users^14^ demonstrated that the older adults engaged in SNSs were more likely to have particular characteristics, such as being female and younger. However, as noted by the authors, SNSs use in older age is a multi-dimensional phenomenon that needs to be understood in the broader context (taking into account aspects such as communication practices, social relationships, individual preferences, etc.). Interestingly, despite the initially relatively small share of older adults among the ICT users, research on online social networking in later life and the associated benefits has been conducted for a longer time (see the literature review on SNSs and older adults by^15^).

The association between social networks and health in older age has been frequently analyzed in both sociological and epidemiological studies: numerous research that were conducted over the last several decades have proven the existence of the association between being a member of/having a social networks and health outcomes, well-being, quality of life (QoL) and mortality, starting from the classic Alameda County Study^16^ and other research conducted in the 1980s^17–19^. Since then, older adults’ social networks and the associations between social networks and health outcomes have been analyzed in many studies (for systematic reviews of studies see:^20–23^; meta-analysis:^24^). Much research has also been devoted to the analyses of the impact of social isolation (an objective state; a lack of a network of social contacts)/social loneliness (a subjective state; perceived social isolation)^25^ on QoL, demonstrating the significant associations between the variables under study (lower level of social loneliness/social isolation – higher QoL) (e.g.^25–28^). In the latter cited study loneliness was negatively associated with both physical and mental QoL (measured with SF-12) among women, and only with mental QoL in men, therefore the authors suggest that loneliness is a gender-specific predictor of QoL^28^. Gender-specific associations of loneliness and other health outcomes were also observed^29^. Gender (along with age and culture) is a factor differentiating the experience of loneliness^30^. Although the results of the studies on gender differences in loneliness across lifespan are sometimes contradictory^31^, women from different cultural contexts commonly express greater level of loneliness in older age than men^32–36^.

There is a growing body of research focused on online social networking of older adults, analyzed in the context of their association with first of all mental health outcomes (for systematic reviews see:^11,37,38^). Many of the studies present internet use^39^ and online social networking as a potential “cure” that helps to alleviate loneliness and social isolation. Five common themes emerging from the papers on the associations between online social networking and mental health outcomes, that were identified in one of the reviews^37^ (i.e. enhanced communication with family and friends; greater independence and self-efficacy; belonging to online communities; positive associations with well-being and life satisfaction; decreased depressive symptoms) seem to adequately summarize the findings from many of the research conducted in this field. However, given the observed trends in using ICT among older adults (in the coming years the number of internet users in the oldest age groups will increase) and taking into account the demographic trends (prolongation of life; dependency and social losses related to the “fourth age”, etc.;^40^) more research is needed about the complexity of the observed associations. It should also be noted that, as emphasized by Fussey and Roth^8^, the ICT use can not only be the source of (social and psychological) benefits, but also bring new threats to well-being (e.g. cyber-crime, surveillance, internet addiction, misinformation, online-induced social loneliness, digital divides perpetuating social inequalities via biased algorithms, etc.). Undoubtedly, both positive and negative impact of ICTs and the use of the SNS requires advanced research^41^.

As mentioned above, the relationship between the level of loneliness and QoL in older age is well established in the literature. However, still little is known about the factors moderating this relationship. In addition to gender^28^, several moderators were identified in research (e.g. SES/housing tenure^42^; interpersonal sensitivity^43^), but the moderating effect of factors such as online social networks is still understudied: one study was found about the moderating role of social networking use on the relationship between loneliness and psychological well-being in a small sample of young adults^44^, but similar analyses conducted on the older age groups is lacking. The intention of our analyses is to fill this gap and provide evidence-based insights to inform public health decision-makers in designing targeted social interventions. Thus the aim of the presented study was to verify the moderating role of personal online social networks in the association between loneliness and QoL in Polish men and women aged 50 years or older.

When starting the analyses, we asked the following research questions:

1. Is there a difference in the level of loneliness between the internet users and non-users aged 50+? If so, is this association differentiated by the way the internet is used (i.e. persons having and not having online social networks) by women and men?
2. Is loneliness associated with QoL in women and men?
3. Does the way the internet is used (i.e. persons having and not having online social networks) moderate the association between the level of loneliness and QoL in men and women aged 50+? In particular: does having online social networks mitigate the association between the level of loneliness and QoL of women and men aged 50+?

The key terms for the presented analyses are conceptualized as follows:

Personal online social networks (or egocentric online social networks) can be defined as a set of the immediate connections/relationships of an individual, established and maintained using ICT, especially (but not exclusively) through the SNS.

Loneliness is a complex and multidimensional construct that cannot be identified with being alone; it involves feelings of isolation, disconnectedness and not belonging/being apart from others^45^; in other words loneliness is the unpleasant experience that occurs when a person’s network of social relations is deficient in some way^46^.

QoL, following the WHO definition, is „the individual’s perception of their position in life in the context of the culture and value systems in which they live and in relation to their goals, expectations, standards and concerns”^47^.

As aging is a long-term process^48^ we believe that examining middle-aged adults alongside older adults can provide insights into its transition phases and allows for identifying early indicators of decline in QoL.

## Methods

### Study design and sampling

The cross-sectional study Research on ageing in Poland—The role of psychosocial determinants in relation to health conditions in ageing process of Polish population (COURAGE - Poland - Comparison After a Decade) (COURAGE-CAD) was conducted in Poland in 2024. There were 2,006 randomly selected community-dwelling individuals from among the general population aged 50+ years. The survey sampling frame was part of the unique Polish personal identification number (PESEL) database. The sample was obtained through a stratified three-stage sampling procedure: (1) Primary Sampling Units (PSUs) were defined as municipalities representing every type of administrative region (rural, small town, medium town, and city) identified in all provinces in Poland, separately for 50 to 64 and 65+ age category. Random sampling of PSUs was done with probability proportional to population size (municipality population size) and with replacement. (2) Secondary Sampling Units were a street (for cities and towns) and a village (for rural areas). These were also sampled with probability proportional to area population size and with replacement. (3) Finally, individual respondents were sampled from SSUs. From each SSU there were 6 respondents sampled (in some cases 7) separately for 50–64 and 65+ age category. Consequently, there were 166 clusters (PSUs), providing approximately 1,000 individual respondents per each age group. To secure the response rate, additional samplings were done. The first and the second replacement sample. Each replacement sample was obtained from the same SSU as it was done for the primary sample, the number of individual respondents was the same, and sampling was done with replacement and also matched by gender. The whole sampling strategy was performed before the beginning of the field study.

Following the AAPOR (2023) guidelines cooperation rate (COOP1) was equal to 52%^49^. The analyzed baseline sample included 1,802 individuals, after excluding proxy respondents due to missing data in the analyzed variables. The study flow chart is presented in Supplementary file 2: Figure S2.1. Informed consent to participate in the study was obtained from each participant.

### Measures

Face-to-face computer-assisted personal interviews using a structured questionnaire were conducted at individuals’ homes by specially trained interviewers. Anthropometric measures were also performed during the interview. Interviewers received a user’s guide for each questionnaire section to standardize data collection. The tools used in this study were previously adapted for the Polish context and validated in COURAGE in Europe study^50^. An additional part was related to online social capital measures.

*QoL* was assessed by the World Health Organization Quality of Life Assessment – Age (WHOQOL-AGE). The tool comprises 13 items focusing on areas which are important for older adults. The final score ranged from 0 to 100 points. Higher scores are interpreted as better QoL. Cultural adaptation and validation properies were described previously^51^. Internal consistency (Cronbach’s alpha) for the study group was 0.93.

*Loneliness* was assessed by the Three-item UCLA Loneliness Scale^45^. The final score ranged from 0 to 100, with higher values indicating a higher level of loneliness. Internal consistency (Cronbach’s alpha) for the study group was 0.88.

### Online social networks (the moderating variables)

At first, there was a brief introduction that the respondents were going to be asked about using the internet, mainly about contacts with other people through the internet. They were asked to consider communication apps (e.g., WhatsApp), social media platforms (e.g., Facebook), internet forums, or communication during playing games on the internet. The question was as follows: *Do you use the internet?* with yes/no answers. The situations where someone else used the internet upon the request of the respondent were marked as *no* in the interviews. Then, to extract the group of people who use the internet not only to maintain their offline social networks, but also to build it through the social network sites (SNSs), communication platforms, etc., participants were asked about being a member of at least one group of people with whom they made connections through the internet (e.g., through the social media platforms, communication apps). Based on this item and the above-mentioned filter question about using the internet, the variable called “internet use and making online social connections” with the following three categories was created: (1) Not using the internet, (2) Using the internet but not a member of online community, (3) Member of online community.

The other considered measure of online social networks was related to frequency of contact with people (beyond work activities) and type of social media used. The respondents were asked about frequency of contacts through the communication apps like WhatsApp or Messenger with 5-point response options (1. *never*, 2. *once or a few times a year*, 3. *once or a few times a month*, 4. *once or a few times a week*, 5. *daily*). Similarly, frequency of contact through social media platforms like Facebook, Instagram, and frequency of contact through emails were measured. Using these three items a cluster analysis (distance measure: Euclidean; clustering method: average) was conducted to identify groups of respondents with similar patterns of behaviors. As a result, a variable called “frequency and type of online social contacts” with the following four categories was created (1) “rare online contact” i.e. on average, they hardly ever use apps and social media, one or few times a year they used emails, (2) “frequent online contact only through apps” i.e. on average, communication apps are used once or a few times a week, while emails are used only once or a few times a year, (3) “frequent online contact through apps and social media platforms, monthly emails” i.e. on average, once or a few times a week they used apps and social media platforms, monthly emails, (4) “frequent online contact through social media platforms, monthly emails, not apps” (for details see Supplementary file 3. Table S3.8). Additionally, the number of people contacted online during the week, the number of people contacted online whom they do not know personally (during the last 12 months), and the number of people they have met on the internet and now know personally. 

Covariates:

#### Demographic and socioeconomic characteristics

Age, place of residence, marital status; objective socioeconomic status (SES) (level of education, individual monthly income in quintiles) and subjective SES—measured at the country level by means of visual analogue scale^52^.

#### Health and functional status

Total number of chronic medical conditions was calculated based on the respondents’ answers about being diagnosed or receiving treatment for chronic lung disease, asthma, arthritis, stroke, cataract, angina, diabetes, cancer, gastrointestinal, kidneys, or oral disease. The presence of depression in the previous 12 months was assessed based on the DSM-IV criteria using an adapted version of the Composite International Diagnostic Interview (CIDI 3.0) ^53,54^. Functioning and disability were assessed using the 12-item version of the World Health Organization Disability Assessment Schedule 2.0 (WHODAS 2.0) and scored on a 0–100 scale with higher scores indicating greater disability^55^. Internal consistency (Cronbach’s alpha) for the study group was 0.94.

The level of social networks was measured by the COURAGE Social Network Index (COURAGE-SNI), which assesses elements of function of social networks (frequency of direct contact, ties, and social support) in eight structural components (spouse or partner, parents, children, grandchildren, other relatives, neighbors, friends, and co-workers). Item Response Theory (IRT) was used to calculate factor scores for five components. One component, related to children, grandchildren, and other relatives, had a hierarchical structure and was referred to as “other family members”. Subsequently, the factor scores were transformed to a 0–100 scale and summed, using the total information provided by IRT for each component as weights. Validity properties and details of the index calculation were described previously^56^.

### Statistical analysis

All data were weighted to account for the sampling design and to generalize the study sample to the reference population. Post-stratification corrections were made to the weights to adjust for non-response. Details are provided in the Supplementary file 1. All estimates of means, proportions, and regression coefficients were computed with design-based (cluster-robust) standard errors.

All analyses were sex-stratified. The comparison of the characteristics at various levels of internet use and making online social connections was conducted using the Chi-squared test, Student’s t-test, Mann-Whitney test, one-way ANOVA, or Kruskal-Wallis test, as appropriate.

To examine moderation effect of “internet use and making online social connections” and “frequency and type of online social contacts” on the association between loneliness and QoL, multiple linear regression models with interaction terms were used. For each moderator, four models were estimated: Model 1 adjusted for age, Model 2 adjusted for age, demographic factors, and socioeconomic status, Model 3 further adjusted for health and functional status, and Model 4 additionally adjusted for the offline Social Network Index. We expected moderation effect, where the interaction terms between loneliness and moderators were found significant (using a significance level of 0.05).

To assess how the effect varies across different levels of the moderators, simple slopes analyses were performed by estimating the conditional effects of loneliness on QoL at specified levels of moderators (using models standardized as described above), followed by visualization of the interaction effects to support interpretation.

Listwise deletion was used to handle missing data on income and subjective economic status in the regression models. Additionally, analyses were conducted on a multiply imputed dataset (see Supplementary file 4).

Data analyses were conducted using IBM SPSS Statistics (Version 29) and R, with the *survey*, *stats*,* emmeans,* and *interactions* packages.

## Results

Our study included 1,038 women and 764 men aged 50 years or older. Men (Me = 57 yrs) were younger than women (Me = 66 yrs), more men were married (72% vs. 59%), and more women had a university degree (18% vs. 14%). There were no significant differences in the percentage of women and men who use the internet, but within the group who use it, women more frequently utilized social media platforms and communication tools (see Supplementary file 3, Table S3.1b).

Basic characteristics of women and men across internet use and making online social connections are presented in Tables 1, 2, and 3. Both men and women who did not use the internet were older, more likely to be widowed, less educated, with lower income and subjective SES, had worse health and functional status, as well as worse QoL in comparison with those who used the internet. In both men and women, members of social-media group were younger, more likely never to have been married, separated, or divorced, had a university degree, and were in the highest income group than those who use the internet but were not members of online community, as well as those who did not use the internet at all. Females who reported membership in an online community showed more frequent depressive symptoms, a higher level of loneliness, and indicated greater disability than those who were not members of such group. In men, contrary to women, no significant differences in the frequency of depressive symptoms as well as the level of loneliness across the compared groups were found. Males who were members of online community indicated the best functional status (Table 2).

**Table 1:** Sociodemographic characteristics of participants by internet use and making online social connections across women (*n* = 1038) and men (*n* = 764), weighted data.

		Women				Men			
		Not using the internet (n^1^ = 423)	Using the internet but not a member of online community (*n* = 532)	Member of online community (*n* = 83)	*p* value	Not using the internet (*n* = 321)	Using the internet but not a member of online community (*n* = 387)	Member of online community (*n* = 56)	*p* value
		%	%	%		%	%	%	
Age (years) Median [Q1;Q3]		71.0 [63.0;77.0]	62.0 [55.0;69.0]	57.0 [54.0;64.0]	*<0.001*	68.0 [62.0;77.0]	61.0 [54.0;67.0]	58.0 [54.0;64.0]	*< 0.001*
Place of residence	Rural	41.2	38.0	40.5	*0.971*	49.3	37.4	42.0	*0.012*
	Urban – < 50 000 inhabitants	25.8	27.8	29.5		23.9	24.0	26.8	
	Urban – 50 000 to 200 000 inh.	18.3	18.5	14.4		16.9	17.4	21.5	
	Urban – > 200 000 inhabitants	14.7	15.6	15.6		9.9	21.2	9.7	
Marital status	Never been married	3.5	4.1	7.4	*< 0.001* ^*W*^	10.8	7.3	15.5	*<0.001* ^*W*^
	Married	45.9	71.2	57.0		64.3	78.0	66.6	
	Separated/Divorced	6.6	8.0	21.8		5.9	6.6	12.4	
	Widowed	44.0	16.7	13.8		19.0	8.2	5.5	
Education level	Primary or lower	31.0	4.9	1.0	*< 0.001*	17.4	4.4	1.1	*<0.001*
	Vocational	39.8	18.8	21.0		58.7	38.4	36.0	
	Secondary	24.8	50.1	34.3		20.6	38.1	27.9	
	Higher	4.4	26.2	43.6		3.3	19.1	35.0	
Individual monthly income in quintiles	≤ 2500 PLN	62.1	33.2	23.7	*< 0.001*	40.9	12.1	10.3	*<0.001*
	2501 – 3500 PLN	22.7	29.3	21.0		26.7	20.8	16.4	
	3501 – 4500 PLN	6.3	16.3	19.3		15.2	22.5	29.8	
	4501 – 6250 PLN	2.9	11.3	16.7		11.4	21.3	16.3	
	> 6250 PLN	6.0	10.0	19.4		5.8	23.3	27.2	
Subjective SES Mean (SD)		5.34 (1.55)	6.40 (1.60)	6.65 (1.70)	*<0.001*	5.60 (1.57)	6.42 (1.48)	6.83 (1.58)	*<0.001*

*Note*: ^1^—unweighted number of respondents; SD - Standard Deviation; Q1–25th Percentile; Q3–75th Percentile; *p value* for the Pearson chi-squared test with second-order Rao-Scott correction, or when appropriate, the adjusted Wald test (W), one-way ANOVA or Kruskal-Wallis test as applicable.

**Table 2:** Health and functional status characteristics, social networks, loneliness and QoL of participants by internet use and making online social connections across women (*n* = 1038) and men (*n* = 764), weighted data.

	Women				Men			
	Not using the internet (n^1^ = 423)	Using the internet but not a member of online community (*n* = 532)	Member of online community (*n* = 83)	*p* value	Not using the internet (*n* = 321)	Using the internet but not a member of online community (*n* = 387)	Member of online community (*n* = 56)	*p* value
Total number of chronic conditions Median [Q1;Q3]	1.0	0.0 [0.0;1.0]	1.0	*< 0.001*	1.0 [0.0;2.0]	0.0 [0.0.;1.0]	0.0 [0.0;1.0]	*0.053*
	[0.0;2.0]		[0.0;1.0]					
*Depression [%]*								
Yes	10.3	6.0	9.3	*0.060*	8.9	4.3	4.9	*0.115* ^*W*^
No	89.7	94.0	90.7		91.1	95.7	95.1	
Functioning and disability (WHODAS 2.0) Median [Q1;Q3]	22.2 [8.3;41.7]	4.2 [0.6;13.6]	6.4 [1.5; 16.1]	*< 0.001*	16.7 [3.1;38.9]	4.2 [0.6;17.8]	2.8 [0.6;13.9]	*< 0.001*
Level of social networks (COURAGE – SNI) Median [Q1;Q3]	57.6 [48.7;65.1]	60.7 [53.7;68.6]	66.8 [58.4;73.5]	*< 0.001*	58.9 [50.7;65.7]	61.0 [52.7;68.2]	62.9 [53.1;69.1]	*0.010*
QoL (WHOQOL-Age) Median [Q1;Q3]	61.5 [50.5;69.5]	72.1 [63.9;77.9]	74.0 [68.3;79.8]	*< 0.001*	62.1 [51.1;70.9]	71.4 [62.7;76.6]	73.6 [65.2;79.4]	*< 0.001*
Loneliness Median [Q1;Q3]	11.1 [0.0;33.3]	0.0 [0.0;22.2]	11.1 [0.0;33.3]	*< 0.001*	0.0 [0.0;33.3]	0.0 [0.0;33.3]	0.0 [0.0;22.2]	*0.801*

*Note*: ^1^—unweighted number of respondents; Q1–25th Percentile; Q3–75th Percentile; *p value* for the Pearson chi-squared test with second-order Rao-Scott correction, or when appropriate, the adjusted Wald test (W) and Kruskal-Wallis test as applicable.

**Table 3:** Online social contacts characteristics of participants by making online social connections across women (*n* = 615) and men (*n* = 443), weighted data.

		Women			Men		
		Using the internet but not a member of online community (n^1^ = 532)	Member of online community (*n* = 83)	*p* value	Using the internet but not a member of online community (*n* = 387)	Member of online community (*n* = 56)	*p* value
		%	%		%	%	
Frequency and type of online social contacts	Rare online contact	14.7	2.3	*<0.001*	26.8	1.6	*< 0.001*
	Frequent online contact only through apps	23.6	1.6		20.9	7.6	
	Frequent online contact through apps and social media platforms, monthly emails	54.0	92.3		45.9	83.5	
	Frequent online contact through social media platforms, monthly emails, not apps	7.7	3.8		6.5	7.4	
Number of Internet contacts per week	Lack of such people	37.0	0.0	*<0.001*	43.1	0.0	*<0.001* ^*W*^
	1 person	3.9	0.9		1.3	0.0	
	2 to 3 people	18.3	11.9		13.7	12.7	
	4 to 5 people	15.5	21.2		15.5	16.6	
	6 to 10 people	16.6	28.5		12.7	14.7	
	11 to 20 people	6.2	21.5		9.3	21.8	
	21 people or more	2.4	16.0		4.4	34.2	
Number of people contacted online and then face-to-face	Lack of such people	88.0	50.8	*<0.001*	80.6	34.3	*< 0.001* ^*W*^
	1 person	4.1	10.0		4.7	16.0	
	2 to 3 people	5.8	22.5		10.4	20.7	
	4 to 5 people	1.1	7.0		1.9	4.1	
	6 people or more	1.0	9.7		2.4	24.9	
Number of people contacted online but not known in person, during the last 12 months	Lack of such people	77.1	29.7	*< 0.001* ^*W*^	65.4	11.6	*<0.001*
	1 person	2.5	5.0		3.4	4.7	
	2 to 3 people	8.6	25.0		15.3	21.0	
	4 to 5 people	6.8	15.9		7.5	13.3	
	6 to 10 people	4.0	14.7		3.5	11.9	
	11 to 20 people	0.5	5.0		0.8	9.5	
	21 people or more	0.6	4.7		4.1	28.0	

*Note*: ^1^—unweighted number of respondents; *p*-value for the Pearson chi-squared test with second-order Rao-Scott correction, or when appropriate, the adjusted Wald test (W).

In both gender groups, members of online community indicated more frequent use of social media platforms, communication apps, as well as emails than non-members (Table S3.2a). Around 70% of them reported that they contacted at least 6 people on the internet (66% of women and 71% of men) in comparison to 25% of female and 26% of male non-members. In women, online community members more frequently indicated that they met in person with someone they had met on the internet (49%- with at least one person) when compared to non-members (12%). The association is also observed in men (66% vs.19% - with at least one person). A lower percentage of female (30%) and male (12%) members of the online community reported that they did not communicate with people they did not know in person, compared to the non-member group (77% and 65%, respectively) (Table 3).

Members of online community also reported the highest level of QoL (Table 2).

### Gender-specific association between loneliness and QoL

The results showed a significant effect of loneliness on QoL among men and women, even in the fully adjusted models (see supplementary Table S3.12).

### Gender-specific moderation effect of “internet use and making online social connections” on the association between loneliness and QoL

In women, the interaction term between loneliness and online social networks (variable 1) was significant even after controlling for sociodemographic variables, health, and functional status, but not for(offline) social networks, despite a marginal p-value (*p* = 0.06) (Table 4). The same analysis conducted on imputed datasets showed significant results also in the fully adjusted model (see supplementary file 4, Table S4.1). In men, no moderation effect was observed (Table 4).

**Table 4:** Linear regression models examining gender-specific moderating role of ‘internet use and making online social connections’ on the association between loneliness and QoL (n = 1802). Weighted data.

Dependent variable: QoL	Model 1		Model 2		Model 3		Model 4	
	β (SE)	*p* value	β (SE)	*p* value	β (SE)	*p* value	β (SE)	*p* value
***Women***								
*Loneliness*	– 0.32 (0.03)	*< 0.001*	– 0.29 (0.03)	*< 0.001*	– 0.20 (0.03)	*< 0.001*	– 0.16 (0.03)	*< 0.001*
*Internet use and making online social connections* (Ref. Not using the internet)								
Using the internet but not a member of online community	7.71 (1.13)	*< 0.001*	4.02 (1.23)	*0.001*	2.88 (1.21)	*0.019*	2.83 (1.14)	*0.015*
Member of online community	7.27 (1.96)	*< 0.001*	2.98 (2.02)	*0.143*	3.39 (1.94)	*0.083*	2.65 (1.99)	*0.187*
Loneliness * Internet use and making online social connections								
Using the internet but not a member of online community	0.01 (0.06)	*0.799*	0.03 (0.05)	*0.503*	– 0.01 (0.05)	*0.759*	– 0.03 (0.05)	*0.592*
Member of online community	0.23 (0.06)	*< 0.001*	0.22 (0.06)	*< 0.001*	0.14 (0.06)	*0.016*	0.11 (0.06)	*0.060*
***Men***								
*Loneliness*	– 0.32 (0.04)	*0.001*	– 0.25 (0.04)	*0.001*	– 0.25 (0.03)	*0.001*	– 0.13 (0.04)	*0.001*
*Internet use and making online social connections* (Ref. Not using the internet)								
Using the internet but not a member of online community	8.33 (1.41)	*0.001*	4.28 (1.54)	*0.007*	4.28 (1.55)	*0.007*	3.35 (1.40)	*0.018*
Member of online community	12.63 (2.30)	*0.001*	7.16 (2.55)	*0.006*	7.16 (2.55)	*0.006*	5.20 (2.13)	*0.016*
Loneliness * Internet use and making online social connections								
Using the internet but not a member of online community	0.02 (0.05)	*0.743*	0.01 (0.05)	*0.789*	0.01 (0.05)	*0.789*	– 0.01 (0.05)	*0.782*
Member of online community	– 0.07 (0.11)	*0.554*	0.01 (0.11)	*0.937*	0.01 (0.11)	*0.937*	– 0.003 (0.08)	*0.968*

*Note*: models examining the moderator’s role in the association between loneliness and QoL; β – unstandardized regression coefficient, SE - standard error; Model 1 - adjusted for age; Model 2 - additionally adjusted for both objective and subjective SES, place of residence and marital status; Model 3 - additionally adjusted for health (presence of depression, total number of chronic conditions, functioning and disability); Model 4 - additionally adjusted for level of social networks.

**Fig. 1:**
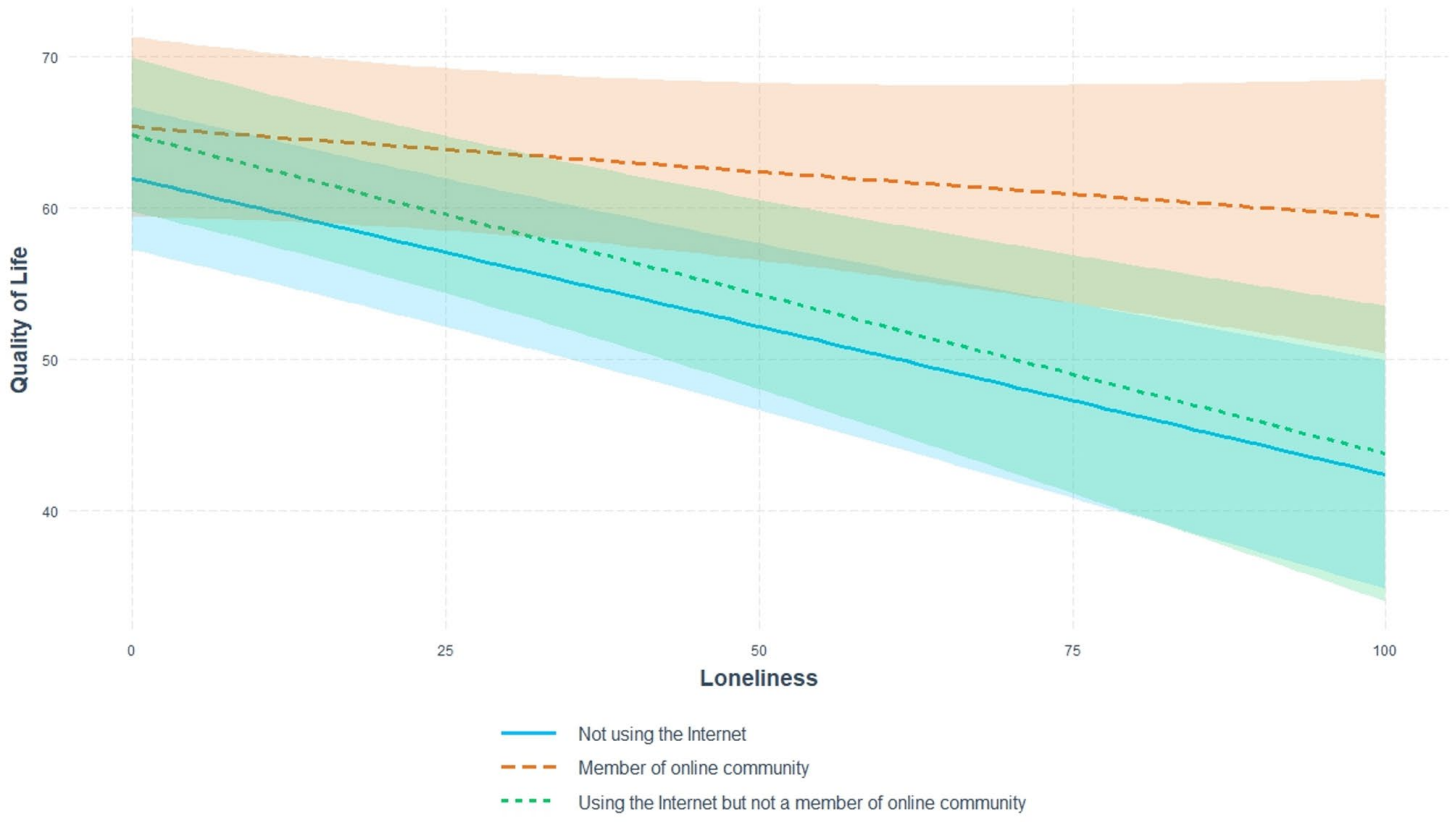
Moderation effect of ‘internet use and making online social connections’ on the association between loneliness and QoL for women (model 3).

The results of the simple slopes analysis showed that among women who were members of online community groups, the association between loneliness and QoL was not statistically significant. Among women who did not use the internet or used it but without being a member of online community higher level of loneliness was associated with lower QoL. The model adjusted only for age, and further models which included additional possible covariates, gave similar results (Supplementary file 3 (Table S3.9) and Fig. 1).

### Gender-specific moderation effect of “frequency and type of online social contacts” on the association between loneliness and QoL

The moderation analysis only among users of the internet was done. In women, the interaction between loneliness and the frequency and type of online social contacts was not statistically significant in the model adjusted only for age. However, the interaction became significant in subsequent models that additionally controlled for sociodemographic variables, health and functional status, and, finally, the offline social networks (Table 5). The same analysis conducted on imputed datasets showed significant results only in model adjusted for age, sociodemographic variables, and health and functional status (see Supplementary file 4, Table S4.2).

**Table 5:** Linear regression models examining the moderating role of ‘frequency and type of online social contacts’ (n = 1058). Weighted data.

Dependent variable: QoL								
	Model 1		Model 2		Model 3		Model 4	
	β (SE)	*p* value	β (SE)	*p* value	β (SE)	*p* value	β (SE)	*p* value
***Women***								
*Loneliness*	– 0.41 (0.08)	*< 0.001*	– 0.41 (0.12)	*< 0.001*	– 0.38 (0.13)	*0.004*	– 0.35 (0.14)	*0.016*
*Frequency and type of online social contacts* (Ref. Rare online contact)								
Frequent online contact only through apps	– 1.76 (2.38)	*0.461*	– 3.79 (2.45)	*0.126*	– 3.97 (2.27)	*0.084*	– 4.13 (2.20)	*0.064*
Frequent online contact through apps and social media platforms, monthly emails	– 1.55 (2.19)	*0.481*	– 3.93 (2.15)	*0.072*	– 2.96 (2.00)	*0.143*	– 3.04 (1.97)	*0.125*
Frequent online contact through social media plaforms, monthly emails, not apps	3.21 (2.85)	*0.262*	1.56 (2.71)	*0.568*	1.98 (2.56)	*0.442*	1.91 (2.55)	*0.458*
Loneliness * Frequency and type of online social contacts (Ref. Rare online contact)								
Frequent online contact only through apps	0.15 (0.10)	*0.156*	0.26 (0.13)	*0.047*	0.3 (0.13)	*0.019*	0.28 (0.13)	*0.035*
Frequent online contact through apps and social media platforms, monthly emails	0.18 (0.10)	*0.074*	0.21 (0.14)	*0.142*	0.21 (0.13)	*0.118*	0.19 (0.14)	*0.192*
Frequent online contact through social media plaforms, monthly emails, not apps	0.18 (0.12)	*0.140*	0.19 (0.14)	*0.187*	0.18 (0.13)	*0.154*	0.17 (0.14)	*0.219*
***Men***								
*Loneliness*	– 0.37 (0.06)	*< 0.001*	– 0.32 (0.07)	*< 0.001*	– 0.22 (0.06)	*< 0.001*	– 0.22 (0.06)	*< 0.001*
*Frequency and type of online social contacts* (Ref. Rare online contact)								
Frequent online contact only through apps	0.68 (2.11)	*0.749*	0.33 (2.54)	*0.897*	1.06 (2.18)	*0.628*	0.52 (2.11)	*0.807*
Frequent online contact through apps and social media platforms, monthly emails	0.92 (1.82)	*0.616*	– 2.46 (2.56)	*0.340*	– 1.38 (2.22)	*0.538*	– 1.8 (2.14)	*0.402*
Frequent online contact through social media plaforms, monthly emails, not apps	0.25 (2.64)	*0.925*	3.05 (3.07)	*0.323*	2.18 (2.72)	*0.427*	1.6 (2.65)	*0.548*
Loneliness * Frequency and type of online social contacts								
Frequent online contact only through apps	0.12 (0.09)	*0.152*	0.09 (0.09)	*0.298*	0.06 (0.09)	*0.504*	0.07 (0.09)	*0.455*
Frequent online contact through apps and social media platforms, monthly emails	0.07 (0.07)	*0.312*	0.13 (0.08)	*0.104*	0.09 (0.07)	*0.249*	0.09 (0.07)	*0.204*
Frequent online contact through social media plaforms, monthly emails, not apps	0.16 (0.11)	*0.156*	0.11 (0.10)	*0.269*	0.06 (0.08)	*0.433*	0.09 (0.08)	*0.239*

*Note*: models examining the moderator’s role in the association between loneliness and QoL; β – unstandardized regression coefficient, SE - standard error; Model 1 - adjusted for age; Model 2 - additionally adjusted for both objective and subjective SES, place of residence and marital status ; Model 3 - additionally adjusted for health (presence of depression, total number of chronic conditions, functioning and disability); Model 4 - additionally adjusted for level of social networks.

In men no significant interaction was observed (Table 5).

**Fig. 2:**
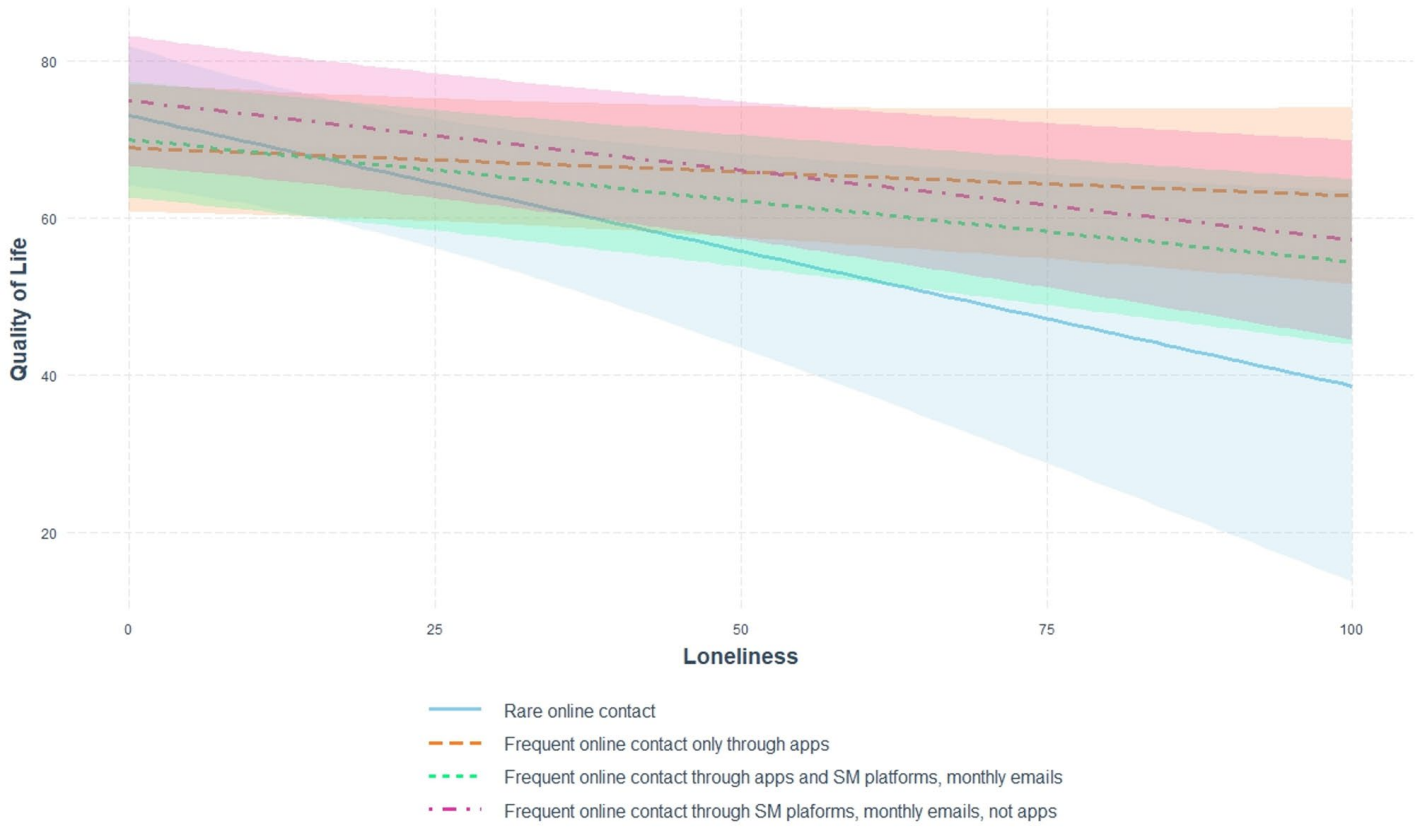
Moderation effect of ‘frequency and type of online social contacts’ on the association between loneliness and QoL for women (model 4).

The results of the simple slopes analysis showed that among women with rare online contact, frequent online contact through both social media and apps, or frequent contact through social media only, higher levels of loneliness were associated with lower QoL. However, among women who frequently used only apps, occasionally emails, but not social media platforms, the effect of loneliness was weaker than in the other groups (model 2) and became statistically insignificant in models additionally adjusted for health and offline social networks (Table S3.10 and Fig. 2).

## Discussion

In response to a critical public health issue related to the need of better understanding the underlying consequences of social isolation and loneliness, this study investigates the role of online social networks as moderators of the impact of loneliness on QoL among middle-aged and older Polish adults. The aim of the current study was triple. The first purpose was to determine if internet use and having personal online social networks differentiates the level of loneliness in middle-aged and older men and women. The second objective was to investigate the link between loneliness and QoL within these gender groups. The third aim was to verify if the way the internet is used moderates the association between loneliness and QoL in middle-aged and older men and women. The latter goal constituted the novelty of the present study.

In relation to the first goal, our findings demonstrate that both men and women who do not use the internet have higher levels of loneliness than those who use it (however in men this relationship is significant only in unweighted data). It is consistent with other studies carried out in different cultural environments and in different age groups. For example, a study on the prevalence of loneliness carried out among individuals aged 16 and over living in the 27 EU member states reveals that exchanging contacts with family members via internet or social media lowers the likelihood of being lonely^57^. Research conducted specifically among older adults also suggests that internet use might be associated with lower loneliness^58,59^, especially when ICTs are used specifically for communication^60^. The way the internet is used further differentiates the levels of loneliness in our sample, but only among women. Those of them who are members of an online network declare higher level of loneliness than those who use the internet but do not participate in an online network. While this finding might be contradictory to expectations, we acknowledge that the cross-sectional nature of our research makes an examination of the direction of causation difficult and that the relationship between loneliness and membership in an online network might be bidirectional and dynamic. It might be that women participate in online social networks in a way that displaces their offline interactions with online activities (instead of forging new friendships and enhancing existing ones), which raises their level of loneliness. However, loneliness – a state associated with interpretative biases and withdrawal – might determine how women interact with SNSs, making them more likely to use them in a way that displaces offline friendships and communications (comp.^60^. This is somehow supported by the results a cross-national study based on a sample of individuals aged 55 + and demonstrating that communicating with family members and close friends primarily via internet, as opposed to non-internet-based forms of contact, is associated with increased loneliness^61^.

With respect to the second aim, our study confirms previous results from different cultural settings on the role of loneliness for the QoL of middle-aged and older adults^28,62–64^. Loneliness substantially decreases QoL of both men and women, and this association remains significant throughout the models, although varies, across age, social-economic-status and health-status group, which demonstrates social inequalities in QoL^65–67^.

We have not observed considerable differences between women and men in the effect of loneliness on QoL throughout the applied models, which is contradictory to some previous research^28^ This might be however due to differences in the study design, measurement tools used, domains of QoL and populations studied. However, recent research^68^ using a constrained multigroup gender model supports our findings through indicating that gender does not significantly alter the relationship between loneliness and QoL.

While our study does not allow us to draw conclusions about the direction of the relationship between loneliness and QoL, studies on longitudinal relationship between these variables confirm that directionality goes from loneliness to QoL^68^ even after accounting for the sociodemographic and health covariates^69^ .

The third aim of our study was to verify whether the way the internet is used moderates the association between the level of loneliness and QoL in men and women aged 50+. The analysis confirms this relationship, by demonstrating that online social networking may alleviate a negative impact of loneliness on middle-aged and older adults’ QoL. However, in our sample this effect is stable only for women. In women, but not in men, the negative impact of loneliness on QoL is buffered by membership in an online community. Particularly, the direct adverse effect of loneliness on QoL disappears in female members of an online community, in comparison to women who do not use the internet and those who use it, however without engaging in an online community. The lack of relationship between these two variables persists after controlling for sociodemographic variables, as well as health and functional status. The significance of the interaction effect falls slightly below the threshold of statistical significance in the fully adjusted model, which includes (offline) social networks; however, the direction of the association remains consistent. Interestingly, the analysis from imputed data shows significant results also in a fully adjusted model. Importantly, the use of the internet itself, without membership in an online community does not protect middle-aged and older adults from the negative effect of loneliness on QoL. This finding is consistent with a prior research on the association of internet use and self-rated health and other health-related outcomes in Polish older adults^70^.

Our findings further suggest that a buffering role in the association between loneliness and QoL is also played by the frequency and type of online contacts. In women, but not in men, frequent social contacts through communication apps significantly reduce the detrimental effect of loneliness in comparison to those who have both rare online contacts and who frequently communicate through social media platforms. This moderating effect holds however at the threshold of significance, and is present only in one model using multiple imputation, and therefore needs further investigation.

Our study supports previous research on the supportive role of online social networks for the QoL of middle-aged and older adults^71^. It goes however further and demonstrates one of the possible mechanisms of this effect through evidencing the mitigation effect of online networking for the adverse impact of loneliness on QoL. To the best of our knowledge, this is the first such study addressing this issue.

The current study also proves that not every form of online contacts plays a protective role, as this effect is significant and stable only for membership in online communities. It also suggests that the relationships under study are gender-specific, as a buffering role of being a member of an online community holds only among women. This result can be interpreted with the reference to the Polish demographic and social context. Due to higher mortality rates observed in men and sex-based differences in life expectancy, women are significantly overrepresented in older age groups and, consequently, are more often widowed (but also divorced) than men^72^. Additionally, big migrations outflows to the Western European countries among younger Poles taking place since the Polish EU accession in 2004^73^ have a further deteriorating impact on middle-aged and older women’s familial social connections. At the same time, as in other countries, women in Poland, including middle-aged women, are better educated than men^74^. Similarly, while the digital skills of the older adults in Poland are lower than in most European countries^75^, in our sample, differently than elsewhere^76^, women use social media and communication tools more often than men. This finding is consistent with recent data on gender differences in internet use^77^ (especially social media platforms use) among older adults in Poland^13^. Additionally, the frequency of internet use is positively correlated with education, which is higher among women^77^. Data also suggest that middle-aged and older women in Poland more often learn new skills, including digital skills, acquired through participation in the universities of the third age^78^. Moreover, a few studies conducted in different national contexts, including Poland, among middle-aged and older adults suggest that women more often use the internet for social purposes and especially to nurture social relationships, while for men it is enough to use the ICTs for practical reasons and to simply stay in touch^79–82^. We can therefore assume that the membership in online social communities allows women to satisfy their needs and create new relationships that replace, or supplement weakened family ties. Women might also use the communication apps to maintain emotional ties with their children and grandchildren who live in a different region or a country^83^.

However, to verify these interpretations it is needed to deepen the analysis by including both quantitative and qualitative characteristics of online networking: not only contact frequency and size, but also density of networks as well with whom the contacts are maintained, existence of emotional bond, and social support. It could be further explored by capturing purposes, for which men and women use the internet and engage in online social networks. Existing research suggests that the purpose of internet use might be a good indicator of at least the psychological impact of online activity. While using the internet for communication proved to positively predict well-being, instrumental internet use was not associated with well-being^84^.

Consistently with previous studies,^59^ our findings suggest that online social networks can compensate for social isolation, which is increasing with age due to lowering physical mobility and loss of family members and friends. It may be because online communities provide – similarly as offline networks – various forms of social support and potentially contribute to self-empowerment and well-being improvement by enabling middle-aged and older women to manage their health conditions better, exchange information and make informed decisions^71,85,86^. As in our analysis the buffering effect of belonging to an online social community on the relationship between loneliness and QoL disappears after standardization on social networks as whole (which is mostly offline), this implies that the use of online social networks can supplement the offline ones when the latter are limited^87^ and defend one’s QoL against the adverse effects of loneliness but cannot alone provide benefits corresponding to all middle-aged and older adults’ needs and totally replace offline face-to-face contacts with family members, friends and colleagues. In the context of demographic changes including migration and family nuclearisation^88,89^, older adults are expected to increasingly rely on remote forms of contacts with their family members and non-family sources of social support, which confirms that both skills and technologies that enable online social contacts become desirable.

The strength of the study was that (1) random sampling approaches was used, with relatively large sample from the whole Poland, where all selected streets or villages were represented by a cluster of respondents; (2) complex indicators of online social networking were applied, capturing not only frequency but also type of social media use as well as allowing for identifying the internet users who actually make online social connections (whereas many studies limit examining the role of online social networks to measuring frequency of going online); (3) wide range of confounding factors were included, such as SES, physical and mental health, functional status and multidimensional instrument to assess all relevant elements of the structure and function of social networks of individuals related to mostly to offline social networks; (4) a loneliness scale measuring three dimensions of the construct under study was used, covering the following: relational connectedness, social connectedness and self-perceived isolation (while many studies employ single-item measures, which demonstrate less reliability and validity than multi-item scales)^32^.

The limitations of the study include (1) cross-sectional study design, thus it was not possible to examine causality; (2) probability of non-response bias, as people with better levels of health and functional status as well as social networks are more likely to participate in this type of study; (3) inclusion of a single national group of middle-aged and older adults in the study; while many of the relationships we found were identified in other studies, cross-national research is necessary for the future to verify our findings on the protective role of online social networks in women in different cultural contexts; (4) focus on the positive aspects of the ICT/SNS use among persons aged 50 + and not addressing potential risks (e.g., misinformation, overuse, etc.), that could bring new threats to QoL of the population under study.

## Conclusions

The study showed that among women (not men) who were members of online community groups the association between loneliness and QoL was not statistically significant in contrary to other internet users and non-users. Among women who frequently used communication only apps, not emails and SNSs, the effect of loneliness was weaker than in the other groups, and was no longer statistically significant in models adjusted for additional covariates. Nonetheless, the later association requires further investigation, since on imputed databset showed signifficant results only in one model. The study showed direction of the possible social intervention to promote healthy aging, especially for women. Encouraging middle-aged and older adult women to join online communities might mitigate the negative effect of loneliness on their QoL. Simultaneously, initiatives aimed at encouraging middle-aged older adults to engage in online social networks should be implemented by trusted organizations that advocate for active and successful aging to mitigate the potential adverse effects of increased social media activity.

## Supplementary Information

Below is the link to the electronic supplementary material.

Supplementary Material 1

Supplementary Material 2

Supplementary Material 3

Supplementary Material 4

## Data Availability

Data will be made available on request. Email: katarzyna.zawisza@uj.edu.pl.

## Abbreviations

ICT: Information and communication technologies
SNS: Social networking site
QoL: Quality of life
SES: Socioeconomic status
