# Supplementary material for: Personal online social networks as moderators of the association between loneliness and quality of life in Polish adults aged 50+
Source: Sci Rep. 2025 Nov 19;15:40797. doi: 10.1038/s41598-025-24545-z (PMC12630643; doi:10.1038/s41598-025-24545-z)
Supplement: Supplementary file 1 — Supplementary Material 1 [file 41598_2025_24545_MOESM1_ESM.docx]

**Personal online social networks as moderators of the association between loneliness and quality of life in Polish adults aged 50+**

Supplementary file 1

**Calculation of survey weights**

**for COURAGE-CAD**

We can consider that two independent samples were drawn in the study, one for each of the age groups. We will calculate the weights for each of the strata independently and create an additional weight when pooled analysis covering all Poland population is performed.

The total study sample was = **2006**.

Table 1. Number of respondents in the study sample across age groups

| **Sample** | **N** | **%** |
| --- | --- | --- |
| 50-64 | 1001 | 49.9 |
| 65+ | 1005 | 50.1 |
| **TOTAL** | **2006** | **100** |

# individual weight

# Weight methodology

Poland was divided in 16 geographical administrative regions (provinces, voivodeships) and 4 categories for administrative region type (size of habitat):

| 1 | rural |
| --- | --- |
| 2 | town <50K inhabitants |
| 3 | town 50-200K inhabitants |
| 4 | town >200K inhabitants |
|  |  |

As a result, 59 strata were created for each age group. In 5 out of 16 provinces (voivodeships), there was no town with a population greater than 200,000 inhabitants.

Name of the voivodeships with no town with a population greater than 200,000 inhabitants:

- lubuskie,
- opolskie,
- podkarpackie,
- świętokrzyskie,
- warmińsko-mazurskie

## Definitions

- PSU (Primary Sampling Unit)= administrative area (municipality )
- SSU (Secondary Sampling Unit)= street or village
- TSU (Tertiary Sampling Unit)= respondent

Table 3. Number of respondents in the study group by geographical administrative regions (voivodeships) and by type of administrative region (size of habitat), with age groups combined

| **voivodeship** | **Strata code (i)** | **Size of habitat** | **N** | **%** | **voivodeship** | **Strata code (i)** | **Size of habitat** | **N** | **%** |
| --- | --- | --- | --- | --- | --- | --- | --- | --- | --- |
| dolnośląskie | **1** | 1 | 44 | (2.2) | podkarpackie | **31** | 1 | 66 | (3.3) |
|  | **2** | 2 | 48 | (2.4) |  | **32** | 2 | 24 | (1.2) |
|  | **3** | 3 | 24 | (1.2) |  | **33** | 3 | 24 | (1.2) |
|  | **4** | 4 | 30 | (1.5) |  | **-** | 4 | - | - |
| kujawsko-pomorskie | **5** | 1 | 42 | (2.1) | podlaskie | **34** | 1 | 24 | (1.2) |
|  | **6** | 2 | 24 | (1.2) |  | **35** | 2 | 13 | (.6) |
|  | **7** | 3 | 27 | (1.3) |  | **36** | 3 | 12 | (.6) |
|  | **8** | 4 | 18 | (.9) |  | **37** | 4 | 12 | (.6) |
| lubelskie | **9** | 1 | 60 | (3.0) | pomorskie | **38** | 1 | 42 | (2.1) |
|  | **10** | 2 | 24 | (1.2) |  | **39** | 2 | 24 | (1.2) |
|  | **11** | 3 | 12 | (.6) |  | **40** | 3 | 9 | (.4) |
|  | **12** | 4 | 18 | (.9) |  | **41** | 4 | 36 | (1.8) |
| lubuskie | **13** | 1 | 18 | (.9) | śląskie | **42** | 1 | 55 | (2.7) |
|  | **14** | 2 | 27 | (1.3) |  | **43** | 2 | 48 | (2.4) |
|  | **15** | 3 | 14 | (.7) |  | **44** | 3 | 114 | (5.7) |
|  | **-** | 4 | - | - |  | **45** | 4 | 30 | (1.5) |
| łódzkie | **16** | 1 | 48 | (2.4) | świętokrzyskie | **46** | 1 | 39 | (1.9) |
|  | **17** | 2 | 30 | (1.5) |  | **47** | 2 | 18 | (.9) |
|  | **18** | 3 | 12 | (.6) |  | **48** | 3 | 12 | (.6) |
|  | **19** | 4 | 42 | (2.1) |  | **-** | 4 | - | - |
| małopolskie | **20** | 1 | 84 | (4.2) | warmińsko-mazurskie | **49** | 1 | 30 | (1.5) |
|  | **21** | 2 | 36 | (1.8) |  | **50** | 2 | 24 | (1.2) |
|  | **22** | 3 | 12 | (.6) |  | **51** | 3 | 19 | (.9) |
|  | **23** | 4 | 42 | (2.1) |  | **-** | 4 | - | - |
| mazowieckie | **24** | 1 | 97 | (4.8) | wielkopolskie | **52** | 1 | 72 | (3.6) |
|  | **25** | 2 | 57 | (2.8) |  | **53** | 2 | 48 | (2.4) |
|  | **26** | 3 | 30 | (1.5) |  | **54** | 3 | 24 | (1.2) |
|  | **27** | 4 | 90 | (4.5) |  | **55** | 4 | 26 | (1.3) |
| opolskie | **28** | 1 | 24 | (1.2) | zachodniopomorskie | **56** | 1 | 25 | (1.2) |
|  | **29** | 2 | 24 | (1.2) |  | **57** | 2 | 30 | (1.5) |
|  | **30** | 3 | 12 | (.6) |  | **58** | 3 | 12 | (.6) |
|  | **-** | 4 | - | - |  | **59** | 4 | 24 | (1.2) |

Table 4. Population count per stratum in Poland (data received from the State System Department, Digital Affairs – Chancellery of the Prime Minister of Poland in 2023)

| **Voivodeship** | **Size of habitat** | **N_i_ (50-64)** | **N_i_ (65+)** | **Voivodeship** | **Size of habitat** | **N_i_ (50-64)** | **N_i_ (65+)** |
| --- | --- | --- | --- | --- | --- | --- | --- |
| dolnośląskie | 1 | 164722 | 154169 | podkarpackie | 1 | 233927 | 206597 |
|  | 2 | 148617 | 188564 |  | 2 | 93441 | 105305 |
|  | 3 | 78581 | 111929 |  | 3 | 62163 | 77802 |
|  | 4 | 94231 | 136813 |  | 4 | 0 | 0 |
| kujawsko-pomorskie | 1 | 155674 | 135972 | podlaskie | 1 | 89402 | 85075 |
|  | 2 | 70943 | 79419 |  | 2 | 54875 | 56303 |
|  | 3 | 79002 | 101734 |  | 3 | 24054 | 24214 |
|  | 4 | 53657 | 76179 |  | 4 | 49606 | 56951 |
| lubelskie | 1 | 207633 | 205953 | pomorskie | 1 | 150455 | 118911 |
|  | 2 | 83133 | 101662 |  | 2 | 94329 | 111596 |
|  | 3 | 32860 | 38919 |  | 3 | 32966 | 41207 |
|  | 4 | 55462 | 72926 |  | 4 | 109086 | 154394 |
| lubuskie | 1 | 65120 | 58884 | śląskie | 1 | 203921 | 186457 |
|  | 2 | 66405 | 79868 |  | 2 | 150011 | 165668 |
|  | 3 | 41944 | 56023 |  | 3 | 362811 | 439757 |
|  | 4 | 0 | 0 |  | 4 | 90297 | 116992 |
| łódzkie | 1 | 170482 | 167839 | świętokrzyskie | 1 | 123916 | 125979 |
|  | 2 | 100619 | 119654 |  | 2 | 55654 | 71783 |
|  | 3 | 53559 | 68693 |  | 3 | 43935 | 61743 |
|  | 4 | 107576 | 163396 |  | 4 | 0 | 0 |
| małopolskie | 1 | 324839 | 279075 | warmińsko-mazurskie | 1 | 109025 | 89119 |
|  | 2 | 119330 | 140247 |  | 2 | 87757 | 99550 |
|  | 3 | 34404 | 40835 |  | 3 | 57377 | 71424 |
|  | 4 | 121143 | 160627 |  | 4 | 0 | 0 |
| mazowieckie | 1 | 347669 | 322128 | wielkopolskie | 1 | 283571 | 248973 |
|  | 2 | 198026 | 225077 |  | 2 | 161136 | 179478 |
|  | 3 | 92203 | 117291 |  | 3 | 77280 | 98237 |
|  | 4 | 266485 | 376566 |  | 4 | 77485 | 113156 |
| opolskie | 1 | 94599 | 81075 | zachodniopomorskie | 1 | 98388 | 86805 |
|  | 2 | 61171 | 72264 |  | 2 | 99927 | 125638 |
|  | 3 | 32831 | 40990 |  | 3 | 27914 | 39977 |
|  | 4 | 0 | 0 |  | 4 | 61549 | 86676 |

Note:

**N_i_ (50-64) -** population size in the i-th stratum aged between 50 and 64 year old

**N_i_ (65+)** - population size in the i-th stratum aged 65 years or older

$m_{i}$ – number of PSU selected in i-th strata in particular age group

$M_{i}^{k}$ – population size in the k-th PSU of the i-th stratum in particular age group

$\mathbf{c}_{\mathbf{k}}$ –number of SSU selected in the k-th PSU in particular age group

$\mathbf{C}_{\mathbf{k}}^{\mathbf{j}}$ – population size in the j-th SSU of the k-th PSU in particular age group

$\mathbf{h}_{\mathbf{j}}$ **–** number of TSU selected in the j-th SSU in particular age group

## **Step 1.** Calculation of the $\mathbf{p}_{\mathbf{1}\mathbf{,}\mathbf{k}}$ **–** probability that the **k-th** PSU in the **i-th** stratum is selected (i.e., the probability of selecting a particular municipality)

$$p_{1,k}=\left\{ \begin{aligned} \frac{M_{i}^{k}}{N_{i}\left( 50-64 \right)}, if the interviewee is aged 50-64 \\ \frac{M_{i}^{k}}{N_{i}\left( 65+ \right)}, if the interviewee is aged 65+..... \end{aligned} \right.$$

## **Step 2.** Calculation of the $\mathbf{p}_{\mathbf{2}\mathbf{,}\mathbf{j}}$ **–** probability that the **j-th** SSU in the **i-th** stratum is selected (i.e., the probability of selecting a particular street/village)

$$p_{2,j}=\left\{ \begin{aligned} c_{k}\cdot\frac{C_{k}^{j}\left( 50-64 \right)}{M_{i}^{k}}, if the interviewee is aged 50-64 \\ c_{k}\cdot\frac{C_{k}^{j}(65+)}{M_{i}^{k}}, if the interviewee is aged 65+..... \end{aligned} \right.$$

## **Step 3.** Calculation of the $\mathbf{p}_{\mathbf{3}}$ **–** probability that the particular person is selected in the **j-th** SSU

$$p_{3}=\left\{ \begin{aligned} \frac{h_{j} (50-64)}{C_{k}^{j}(50-64)}, if aged 50-64 \\ \frac{h_{j} (65+)}{C_{k}^{j}(65+)}, if aged 65+..... \end{aligned} \right.$$

## ***Step 4.*** Final „probability”:

$$\boldsymbol{p}_{\boldsymbol{f}}\boldsymbol{=}\boldsymbol{p}_{\boldsymbol{1,k}}\boldsymbol{\cdot}\boldsymbol{p}_{\boldsymbol{2,j}}\boldsymbol{\cdot}\boldsymbol{p}_{\boldsymbol{3}}$$

## ***Step 5.*** Final weight:

$$\boldsymbol{w}_{\boldsymbol{f}}\boldsymbol{=}\frac{\boldsymbol{1}}{\boldsymbol{p}_{\boldsymbol{f}}}$$

The sum of $\mathbf{w}_{\mathbf{f}}$ should be the total population in each considered age sample of Poland. Final weights were adjusted to ensure that the sum of W_f_ in each sample corresponds to the actual population size in each age group.

|  | Sum ($\mathbf{w}_{\mathbf{f}}$) | Population of Poland* |
| --- | --- | --- |
| $\mathbf{w}_{\mathbf{f}}\boldsymbol{(}$50-64) | 6 369 977.88 | 6 689 190 |
| $\mathbf{w}_{\mathbf{f}}$ (65+) | 6 927 786.10 | 7 420 538 |

*The size of the Polish population at the time of participant selection was used (2023).

The following formula was calculated for each age sample

$$\boldsymbol{w*}_{\boldsymbol{f}}\boldsymbol{=}\frac{\boldsymbol{Population of Poland}}{\boldsymbol{Sum(}\boldsymbol{w}_{\boldsymbol{f}}\boldsymbol{)}}\boldsymbol{\cdot}\boldsymbol{w}_{\boldsymbol{f}}$$

1. **Post- stratification weight**

The post-stratification is used to obtain the same distribution of sex and age in the sample survey and population. Weight was rescaled to achieve a mean value of one,

WPST3$=\left\{ \begin{aligned} \frac{1001}{6 689 190}, if aged 50-64 \\ \frac{1005}{7 420 538}, if aged 65+...... \end{aligned} \right.$

$$WPost=\boldsymbol{w*}_{\boldsymbol{f}}\boldsymbol{\cdot WPST}\boldsymbol{3}$$

Descriptive statistics of individual post-stratification weight

|  | Mean | | SD | Minimum | Maximum | Median | Sum |
| --- | --- | --- | --- | --- | --- | --- | --- |
| WPost_50_64 | 1.00 | 0.39 | | 0.24 | 6.22 | 1.03 | 1001 |
| WPost_65 | 1.00 | 0.36 | | 0.24 | 3.42 | 1.01 | 1005 |

Next tables show population and the sample distribution of sex in each age group (50-64 and 65+).

| $\boldsymbol{Population of Poland}$ | | | |  | **In the survey, after weighting by WPost** | | | |
| --- | --- | --- | --- | --- | --- | --- | --- | --- |
| N | **50-64** | **65+** | total |  | N | **50-64** | **65+** | total |
| **Women** | 3 480 853 | 4 449 777 | 7 930 630 |  | **Women** | 586 | 564 | 1150 |
| **Men** | 3 208 337 | 2 970 761 | 6 179 098 |  | **Men** | 415 | 441 | 856 |
| Suma | 6 689 190 | 7 420 538 | 14 109 728 |  | Suma | 1001 | 1005 | 2006 |

| $\boldsymbol{Population of Poland}$ | | | |  | **In the survey, after weighting by WPost** | | | |
| --- | --- | --- | --- | --- | --- | --- | --- | --- |
| % | **50-64** | **65+** | total |  | % | **50-64** | **65+** | total |
| **Women** | 52.04 | 59.97 | 56.21 |  | **Women** | 58.54 | 56.12 | 57.33 |
| **Men** | 47.96 | 40.03 | 43.79 |  | **Men** | 41.46 | 43.88 | 42.67 |

The final post-stratification weight is obtained by calculating the sex factor in each sample, as follows:

$$F_{sex}=\left\{ \begin{aligned} \frac{proportion of males in population}{proportion of males in sample survey}, for males \\ \frac{proportion of females in population}{proportion of females in sample survey}, for females........ \end{aligned} \right.$$

$$F_{sex}=\left\{ \begin{aligned} \frac{52.04}{58,54}=0.88896, for females aged 50-64 \\ \frac{59.97}{56.12}=1.06860, for females aged 65+... \end{aligned} \right.$$

$$F_{sex}=\left\{ \begin{aligned} \frac{47.96}{41.46}= 1.15677, for males aged 50-64 \\ \frac{40.03}{43.88}=0.91226 , for males aged 65+... \end{aligned} \right.$$

Rescaling weight, to obtain the same distribution of sex in the sample survey and population.:

$$\boldsymbol{WPost\_final=}\boldsymbol{WPost\cdot}\boldsymbol{F}_{\boldsymbol{sex}}$$

| **The distribution of sex in age groups after weighting by WPost_final** | | | | | |
| --- | --- | --- | --- | --- | --- |
|  | **50-64** | | **65+** | | **total** |
|  | N | **%** | N | **%** | **%** |
| **Women** | 521 | **52.04** | 602 | **59.95** | **55.99** |
| **Men** | 480 | **47.96** | 403 | **40.05** | **44.01** |

Weight independent for age group (50+):

|  | **50-64** | **65+** | total |
| --- | --- | --- | --- |
| Population of Poland | 6 689 190 | 7 420 538 | 14 109 728 |
| % | 47.41 | 52.59 | 100 |
| Study sample | 1001 | 1005 | 2006 |
| % | 49.9 | 50.1 | 100 |

$$Coef=\left\{ \begin{aligned} \frac{proportion in population}{estimated proportion in sample survey}, if aged 50-64 \\ \frac{proportion in population}{estimated proportion in sample survey}, if aged 65+.. \end{aligned} \right.$$

$$Coef=\left\{ \begin{aligned} \frac{47.41}{49.9}=0.95010, if aged 50-64 \\ \frac{52.59}{50.1}=1.04970, if aged 65+ . \end{aligned} \right.$$

Finally, the normalized individual post-stratification weight was calculated

$$\boldsymbol{WPost\_country=}\boldsymbol{WPost\_final\cdot Coef}$$

## Descriptive statistics (n=2006)

Descriptive statistics of weight variable ( WPost_country variable) are as follows:

|  | Mean | SD | Min | Max | Median | Sum |
| --- | --- | --- | --- | --- | --- | --- |
| **WPost_country** | 1.00 | 0.39 | 0.21 | 6.84 | 1.01 | 2005.96 |

1. **Weight variable for data base after proxy respondents exclusion (n=1802)**

We need to return to paragraph II and, using the same method, calculate the following coefficients based on a sample of 1802 respondents: **WPST3**, **F_sex**, and **Coef**, in order to obtain the weight variable.

Descriptive statistics of weight variable ( WPost_country_2 variable), are as follows:

|  | Mean | SD | Min | Max | Median | Sum |
| --- | --- | --- | --- | --- | --- | --- |
| **WPost_country_2** | 1.00 | 0.40 | 0.21 | 6.87 | 0.98 | 1802.00 |
