# Supplementary material for: Personal online social networks as moderators of the association between loneliness and quality of life in Polish adults aged 50+
Source: Sci Rep. 2025 Nov 19;15:40797. doi: 10.1038/s41598-025-24545-z (PMC12630643; doi:10.1038/s41598-025-24545-z)
Supplement: Supplementary file 2 — Supplementary Material 2 [file 41598_2025_24545_MOESM2_ESM.docx]

**Personal online social networks as moderators of the association between loneliness and quality of life in Polish adults aged 50+**

Supplementary file 2

**Study design and number of participants**

**for COURAGE-CAD**

Spis treści

[Figure S2.1. Flow chart 2](#_Toc207802349)

[Cooperation rate 3](#_Toc207802350)

[Table S2.2. Number of selected municipalities (stage 1. ) 4](#_Toc207802351)

[**Figure S2.2 Participant count by province (voivodeship)** 6](#_Toc207802352)

**Stage 2.** A total of 598 various Secondary Sampling Units (SSUs) were finally selected (in case of selection of street or village with insufficient number of respondents additional streets/villages were randomly chosen in the same PSU).

**Stage 1.** Randomly selected 266 various municipalities which is related to assumed 332 clusters – Primary Sampling Units (PSUs).

\\\

Additionally, a fourth sample of respondents was randomly selected in the same manner to serve as a reserve, used only if—after at least three contact attempts across all three initial samples—it was not possible to conduct six interviews. In total, 370 contacts were drawn from the reserve sample

**Stage 3.** 6004 randomly selected respondents 🡪 2000 respondents were randomly selected (6 per cluster, in a few cases 7 per cluster) with two additional respondents from the same SSU (matched additionally by gender) (2000 – main sample, 2000 the first replacement sample, 2004 – the second replacement sample). 997 contacts not used.

\

**Total COURAGE-CAD study sample** (n=2006, 37.3%)

**COOP1=52%** (see formula below)

**Non-contact (NC)** (n=1279 (37.94%))

- Respondent is absent/no one opens the door

**Other (O)** (n=3 (0.09%))

- Language barrier

**Nonresponse** (n=3371, 62.7%)

Dropouts (n=3282)

**Non-contact (NC) (n=1279 (38.97%))**

- Respondent is absent/no one opens the door (n=1279 (24.19%))

Excluded proxy interviews (n=204 (10.2%))

Respondents (n=1802 (89.8%))

**Respondents**  (n=1802 (89.9%))

**Excluded proxy interviews** (n=204 (10.2%))

No information about quality of life and loneliness was gathered due to the need for subjective assessment by the respondents.

**Unknown if household (UH)** (n=39 (1.16%))

- Region is unsafe or inaccessible to respondent’s household (address not available) (n=11 (0.33%))
- Invalid address, address doesn’t exist/uninhabited address (vacant property, commercial premises) (n=28 (0.83%))

**Unknown, other (UO)** (n=214 (6.35%))

- Death of respondent (n=43 (1.28%))
- No possibility to obtain information about the respondent (no one at the address indicated has information about the respondent (n=43 (1.28%))
- Change of respondent’s place of residence (n=67 (1.99%)
- The respondent doesn’t belong to the target group (n=6 (0.18%))
- The respondent has moved abroad (n=50 (1.48%))
- Interview excluded because the cluster contained too many interviews (n=5 (0.15%))

**Refusal and break-off (R)** (n=1836 (54.46%))

- Partial interview (interview conducted partially, respondent doesn’t want to be conducted in future (n=2 (0.06%))
- Definitive refusal from respondent (n=1764 (52.33%))
- No interview, because respondent is unable to respond due to mental limitation or severe disease and the proxy respondent refuses to participate in the survey (n=27 (0.80%))
- Abstention/refusal by proxy respondent (n=43 (1.28%))

### Figure S2.1. Flow chart

Dropouts (n=3282)

**Refusal and break off (R) (n=1836 (55.94%))**

- Partial interview (interview conducted partially, respondent doesn’t want to be conducted in future (n=2 (0.04%))
- Definitive refusal from respondent (n=1764 (33.6%))
- No interview, because respondent is unable to respond due to mental limitation or severe disease and the proxy respondent refuses to participate in the surveys (n=27 (0.51%))
- Abstention/refusal by proxy respondent (n=43 (0.81%))

**Non-contact (NC) (n=1279 (38.97%))**

- Respondent is absent/no one opens the door (n=1279 (24.19%))

**Total COURAGE-CAD study sample (n=2006)**

Excluded proxy interviews (n=204 (10.2%))

Respondents (n=1802 (89.8%))

Dropouts (n=3282)

**Non-contact (NC) (n=1279 (38.97%))**

- Respondent is absent/no one opens the door (n=1279 (24.19%))

Excluded proxy interviews (n=204 (10.2%))

Respondents (n=1802 (89.8%))

### Cooperation rate

To examine the scale of refusal and to infer about the level of willingness to participate in the COURAGE-CAD study, we calculated cooperation rate.

$$COOP1=\frac{I}{I+R+O}$$

The cooperation rate 1 (COOP1), minimum cooperation rate is proportion the complete number of interviews (I) to the sum of complete number of interviews (I) and the number of non-interviews with all eligible units ever contacted (refusal and break-off (R) and other (O)).

Reference: AAPOR. Response Rates Calculator. https://aapor.org/response-rates/ (2023).

### Table S2.2. Number of selected municipalities (stage 1. )

| Name of the geographical administrative regions (voivodships) | size of habitat (1. rural, 2. urban(<50k inh.), 3. urban( 50-200k inh.), 4.urban (>200k inh.) | number of clusters to select (municipalities^1^) | | number of selected municipalities (considering replacement) | |
| --- | --- | --- | --- | --- | --- |
|  |  | 50-64 yrs | 65+ yrs | 50-64 yrs | 65+ yrs |
| dolnośląskie | 1 | 4 | 3 | 4 | 3 |
| dolnośląskie | 2 | 4 | 4 | 4 | 4 |
| dolnośląskie | 3 | 2 | 2 | 2 | 2 |
| dolnośląskie | 4 | 2 | 3 | 1 | 2 |
| kujawsko-pomorskie | 1 | 4 | 3 | 4 | 3 |
| kujawsko-pomorskie | 2 | 2 | 2 | 2 | 2 |
| kujawsko-pomorskie | 3 | 2 | 2 | 2 | 2 |
| kujawsko-pomorskie | 4 | 1 | 2 | 1 | 1 |
| lubelskie | 1 | 5 | 5 | 5 | 5 |
| lubelskie | 2 | 2 | 2 | 2 | 2 |
| lubelskie | 3 | 1 | 1 | 1 | 1 |
| lubelskie | 4 | 1 | 2 | 1 | 1 |
| lubuskie | 1 | 2 | 1 | 2 | 1 |
| lubuskie | 2 | 2 | 2 | 2 | 2 |
| lubuskie | 3 | 1 | 1 | 1 | 1 |
| mazowieckie | 1 | 9 | 7 | 9 | 6 |
| mazowieckie | 2 | 5 | 5 | 4 | 4 |
| mazowieckie | 3 | 2 | 3 | 2 | 2 |
| mazowieckie | 4 | 7 | 8 | 6 | 8 |
| małopolskie | 1 | 8 | 6 | 7 | 6 |
| małopolskie | 2 | 3 | 3 | 3 | 3 |
| małopolskie | 3 | 1 | 1 | 1 | 1 |
| małopolskie | 4 | 3 | 4 | 2 | 2 |
| opolskie | 1 | 2 | 2 | 2 | 2 |
| opolskie | 2 | 2 | 2 | 2 | 2 |
| opolskie | 3 | 1 | 1 | 1 | 1 |
| podkarpackie | 1 | 6 | 5 | 6 | 5 |
| podkarpackie | 2 | 2 | 2 | 2 | 2 |
| podkarpackie | 3 | 2 | 2 | 1 | 2 |
| podlaskie | 1 | 2 | 2 | 2 | 2 |
| podlaskie | 2 | 1 | 1 | 1 | 1 |
| podlaskie | 3 | 1 | 1 | 1 | 1 |
| podlaskie | 4 | 1 | 1 | 1 | 1 |
| pomorskie | 1 | 4 | 3 | 4 | 2 |
| pomorskie | 2 | 2 | 2 | 2 | 2 |
| pomorskie | 3 | 1 | 1 | 1 | 1 |
| pomorskie | 4 | 3 | 3 | 2 | 2 |
| warmińsko-mazurskie | 1 | 3 | 2 | 3 | 2 |
| warmińsko-mazurskie | 2 | 2 | 2 | 2 | 2 |
| warmińsko-mazurskie | 3 | 1 | 2 | 1 | 1 |
| wielkopolskie | 1 | 7 | 5* | 7 | 5 |
| wielkopolskie | 2 | 4 | 4 | 4 | 4 |
| wielkopolskie | 3 | 2 | 2 | 2 | 2 |
| wielkopolskie | 4 | 2 | 2* | 2 | 2 |
| zachodniopomorskie | 1 | 2 | 2 | 2 | 2 |
| zachodniopomorskie | 2 | 2 | 3 | 2 | 3 |
| zachodniopomorskie | 3 | 1 | 1 | 1 | 1 |
| zachodniopomorskie | 4 | 2 | 2 | 1 | 1 |
| łódzkie | 1 | 4 | 4 | 4 | 4 |
| łódzkie | 2 | 2* | 3 | 2 | 3 |
| łódzkie | 3 | 1 | 1* | 1 | 1 |
| łódzkie | 4 | 3 | 4 | 2 | 3 |
| śląskie | 1 | 5 | 4 | 5 | 4 |
| śląskie | 2 | 4 | 4 | 4 | 4 |
| śląskie | 3 | 9 | 10 | 8 | 9 |
| śląskie | 4 | 2 | 3 | 1 | 1 |
| świętokrzyskie | 1 | 3 | 3 | 2 | 3 |
| świętokrzyskie | 2 | 1 | 2 | 1 | 1 |
| świętokrzyskie | 3 | 1 | 1 | 1 | 1 |
|  |  | *additional cluster was selected to achieve assumed 1000 people/age group | *additional respondents were randomly selected on the final stage to achieve assumed 1000 people/age group |  |  |

inh.- inhabitants;^1^ – or city districts (if they have a unique territorial code)

### **Figure S2.2 Participant count by province (voivodeship) across age and gender groups**

| 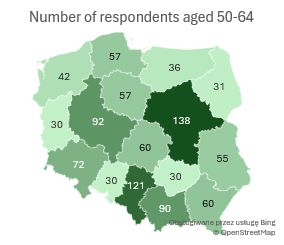 | 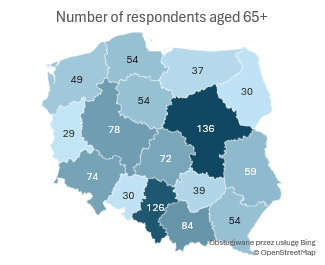 |
| --- | --- |
| 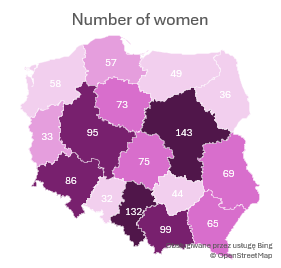 | 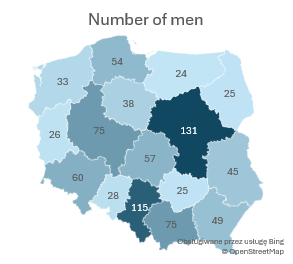 |

### **R code for study design, *survey* package :**

baza$strata <- interaction(baza$province, baza$municipality_type, baza$age_group, drop=TRUE) # Baza – name of dataset

options(survey.lonely.psu="adjust")

design <- svydesign(

ids = ~municipality+ town_village + street,

strata = ~strata,

weights = ~WPost_country_2,

data = baza,

nest=TRUE

)
