# Supplementary material for: Personal online social networks as moderators of the association between loneliness and quality of life in Polish adults aged 50+
Source: Sci Rep. 2025 Nov 19;15:40797. doi: 10.1038/s41598-025-24545-z (PMC12630643; doi:10.1038/s41598-025-24545-z)
Supplement: Supplementary file 3 — Supplementary Material 3 [file 41598_2025_24545_MOESM3_ESM.docx]

**Personal online social networks as moderators of the association between loneliness and quality of life in Polish adults aged 50+**

Supplementary file 3

Table of contents

[Figure S3.1. The selection of participants of the COURAGE-CAD study. 1](#_Toc1135861708)

[Descriptive statistics 2](#_Toc1140201453)

[Table S3.1a Characteristics of participants by gender, unweighted data (n=1802). 3](#_Toc457212503)

[Table S3.1b Characteristics of participants by gender, weighted data (n=1802). 4](#_Toc814348321)

[Table S3.2a Frequencies of using SM platform, communication tools and emails in the last 12 months, unweighted data. 6](#_Toc1936074622)

[Table S3.3a Frequencies of using SM platform (e.g. Facebook, Instagram, X) for contacts with family, friends and acquaintances among women and men (n=1058), unweighted data. 8](#_Toc785369605)

[Table S3.3b Frequencies of using SM platform (e.g. Facebook, Instagram, X) for contacts with family, friends and acquaintances among women and men (n=1058), weighted data. 10](#_Toc1228049267)

[Table S3.4a Frequencies of contact using communication tools (e.g., WhatsApp, Messenger, Zoom, Skype) with family, friends and acquaintances among women and men (n=1058), unweighted data. 12](#_Toc2047209350)

[Table S3.4b Frequencies of contact using communication tools (e.g., WhatsApp, Messenger, Zoom, Skype) with family, friends and acquaintances among women and men (n=1058), weighted data. 14](#_Toc380171496)

[Table S3.5. Sociodemographic characteristics of participants by internet use and making online social connections across women (n=1038) and men (n=764), unweighted data. 16](#_Toc705473540)

[Table S3.6. Health and functional status characteristics, social networks, loneliness and QoL of participants by internet use and making online social connections across women (n=1038) and men (n=764), unweighted data. 17](#_Toc99317112)

[Table S3.7. Online social contacts characteristics of participants by making online social connections across women (n=615) and men (n=443), unweighted data. 18](#_Toc1900139507)

[Cluster analysis 20](#_Toc37143447)

[Table S3.8. Cluster means for social media, communication apps, and email use frequencies for each cluster solution. 20](#_Toc1847173640)

[Regression models 21](#_Toc87418129)

[Table S3.9. Simple Slopes Analysis: Conditional Effects of Loneliness on Quality of Life Across Internet Use and Making Online Social Connections for Women. Weighted data. 21](#_Toc267466515)

[Table S3.10. Simple Slopes Analysis: Conditional Effects of Loneliness on Quality of Life Across Frequency and Type of Online Social Contacts for women (model 4). Weighted data. 21](#_Toc490527309)

[Table S3.11. Linear regression models: associations between loneliness and QoL, without interactions. Unweighted data. 22](#_Toc1528525838)

[Table S3.12. Linear regression models: associations between loneliness and QoL, without interactions. Weighted data. 23](#_Toc1372695203)

### Figure S3.1. The selection of participants of the COURAGE-CAD study.

Excluded proxy interviews

due to missing data, particularly in quality of life and loneliness measures (n=204 (10.2%))

Men (n= 96) Women (n=108)

Internet users

(n=1058 (58.7%))

Men (n=443) Women (n=615)

Non-internet users

(n=744 (41.3%))

Men (n=321) Women (n=423)

Number of missing values in any of the analyzed variables:

Individual monthly income (n=224)

Subjective SES (n=44)

Functioning and disability (n=0, n=684*,)

Number of missing values in any of the analyzed variables:

Individual monthly income (n=154)

Subjective SES (n=60)

Functioning and disability (n=0, n=450*)

Total COURAGE-CAD sample

(n=2006)

Respondents

(n=1802 (89.8%))

Men (n=764) Women (n=1038)

^*^Missing data values for one of 12 items were handled using multiple imputation with monotone method, based on the complete items of the WHODAS 2.0 scale.

## Descriptive statistics

### Table S3.1a Characteristics of participants by gender, unweighted data (n=1802).

|  | |  | **Women** (n=1038) | **Men**  (n=764) | *p value* | |
| --- | --- | --- | --- | --- | --- | --- |
|  | |  | n (%) | n (%) |  |  |
| Age (years)  Median [Q1;Q3] | | | 63.00 [57.00;72.00] | 64.00 [57.00;71.00] | *0.890* |  |
| Place of residence | Rural | | 397 (38.2) | 303 (39.7) | *0.683* |  |
|  | Urban – less than 50 000 inhabitants | | 266 (25.6) | 177 (23.2) |  |  |
|  | Urban – 50 000 to 200 000 inhabitants | | 187 (18.0) | 139 (18.2) |  |  |
|  | Urban – more than 200 000 inhabitants | | 188 (18.1) | 145 (19.0) |  |  |
| Marital status | Never been married | | 48 (4.6) | 70 (9.2) | *<0.001* |  |
|  | Married | | 620 (59.7) | 550 (72.0) |  |  |
|  | Separated/Divorced | | 93 (9.0) | 46 (6.0) |  |  |
|  | Widowed | | 277 (26.7) | 98 (12.8) |  |  |
| Education level | Primary or lower | | 149 (14.4) | 71 (9.3) | *<0.001* |  |
|  | Vocational | | 292 (28.1) | 350 (45.8) |  |  |
|  | Secondary | | 401 (38.6) | 236 (30.9) |  |  |
|  | University | | 196 (18.9) | 107 (14.0) |  |  |
| Individual monthly income in quintiles | ≤ 2 500 PLN | | 340 (41.8) | 146 (23.9) | *<0.001* |  |
|  | 2 501 – 3 500 PLN | | 207 (25.4) | 135 (22.1) |  |  |
|  | 3 501 – 4 500 PLN | | 111 (13.6) | 125 (20.5) |  |  |
|  | 4 501 – 6 250 PLN | | 71 (8.7) | 105 (17.2) |  |  |
|  | >6 250 PLN | | 85 (10.4) | 99 (16.2) |  |  |
| Subjective SES  Mean (SD) | | | 6.01 (1.67) | 6.17 (1.61) | *0.042* |  |
| Level of social networks (COURAGE - SNI)  Median [Q1;Q3] | | | 59.82 [52.07;68.09] | 60.24 [51.95;67.28] | *0.635* |  |
| Total number of chronic conditions  Median [Q1;Q3] | | | 1.00 [0.00;1.00] | 0.00 [0.00;1.00] | *0.020* |  |
| Depression | Yes | | 83 (8.0) | 47 (6.2) | *0.161* |  |
|  | No | | 955 (92.0) | 717 (93.8) |  |  |
| Functioning and disability (WHODAS 2.0)  Median [Q1;Q3] | | | 8.33 [1.11;25.56] | 7.78 [0.83;25.00] | *0.122* |  |
| Quality of life (WHOQOL-Age)  Mean (SD) | | | 66.09 (15.23) | 66.44 (15.38) | *0.635* |  |
| Loneliness  Median [Q1;Q3] | | | 0.00 [0.00;33.33] | 0.00 [0.00;33.33] | *0.552* |  |
| Using Internet | Yes | | 615 (59.2) | 443 (58.0) | *0.624* |  |
|  | No | | 423 (40.8) | 321 (42.0) |  |  |
| Frequency of using social media platforms (e.g., Facebook, Instagram, X) | Never | | 190 (30.9) | 184 (41.5) | *0.002* |  |
|  | Once or a few times a year | | 20 (3.3) | 13 (2.9) |  |  |
|  | Once or a few times a month | | 84 (13.7) | 41 (9.3) |  |  |
|  | Once or a few times a week | | 173 (28.1) | 96 (21.7) |  |  |
|  | Daily | | 148 (24.1) | 109 (24.6) |  |  |
| Frequency of using communication tools (e.g., WhatsApp, Messenger, Zoom, Skype) | Never | | 106 (17.2) | 116 (26.2) | *0.005* |  |
|  | Once or a few times a year | | 20 (3.3) | 14 (3.2) |  |  |
|  | Once or a few times a month | | 69 (11.2) | 32 (7.2) |  |  |
|  | Once or a few times a week | | 171 (27.8) | 114 (25.7) |  |  |
|  | Daily | | 249 (40.5) | 167 (37.7) |  |  |
| Frequency of sending/receiving emails in the last 12 months | Never | | 211 (34.3) | 174 (39.3) | *0.142* |  |
|  | Once or a few times a year | | 106 (17.2) | 54 (12.2) |  |  |
|  | Once or a few times a month | | 124 (20.2) | 81 (18.3) |  |  |
|  | Once or a few times a week | | 94 (15.3) | 72 (16.3) |  |  |
|  | Daily | | 80 (13.0) | 62 (14.0) |  |  |
| Frequency and type of online social contacts | Rare online contact | | 86 (19.1) | 101 (22.8) |  |  |
|  | Frequent online contact only through apps | | 113 (18.4) | 89 (20.1) | *<0.001* |  |
|  | Frequent online contact through apps and social media platforms, monthly emails | | 371 (60.3) | 223 (50.3) |  |  |
|  | Frequent online contact through social media platforms, monthly emails, not apps | | 45 (7.3) | 30 (6.8) |  |  |

*Note*: SD - Standard Deviation; Q1- 25th Percentile; Q3 - 75th Percentile; *p value* for the Mann-Whitney test, Chi-squared test of independence or Student's t-test, as applicable

### Table S3.1b Characteristics of participants by gender, weighted data (n=1802).

|  | |  | **Women** (n=1038) | **Men** (n=764) |  |
| --- | --- | --- | --- | --- | --- |
|  |  |  |  |  |  |
|  | |  | % | % | *p value* |
| Age (years) | | | 66.00 [58.00; 73.00] | 57.00 [64.00;71.00] | *0.001* |
| Median [Q1;Q3] | | |  |  |  |
| Place of residence | Rural | | 39.6 | 42.6 | *0.505* |
|  | Urban – less than 50 000 inhabitants | | 27.1 | 24.2 |  |
|  | Urban – 50 000 to 200 000 inhabitants | | 18.1 | 17.5 |  |
|  | Urban – more than 200 000 inhabitants | | 15.2 | 15.7 |  |
| Marital status | Never been married | | 4.1 | 9.3 | *<0.001* |
|  | Married | | 59.2 | 71.6 |  |
|  | Separated/Divorced | | 8.5 | 6.7 |  |
|  | Widowed | | 28.3 | 12.4 |  |
| Education level | Primary or lower | | 15.9 | 9.4 | *<0.001* |
|  | Vocational | | 28.0 | 46.5 |  |
|  | Secondary | | 38.0 | 30.2 |  |
|  | University | | 18.2 | 13.9 |  |
| Individual monthly income in quintiles | ≤ 2 500 PLN | | 44.5 | 23.9 | *<0.001* |
|  | 2 501 – 3 500 PLN | | 25.8 | 22.9 |  |
|  | 3 501 – 4 500 PLN | | 12.4 | 20.1 |  |
|  | 4 501 – 6 250 PLN | | 8.2 | 16.8 |  |
|  | >6 250 PLN | | 9.2 | 16.4 |  |
| Subjective SES | | | 5.97 (1.68) | 6.13 (1.58) | *0.062* |
| Mean (SD) | | |  |  |  |
| Level of social networks (COURAGE - SNI) | | | 59.67 [51.72;67.75] | 59.87 [51.86;66.97] | *0.708* |
| Median [Q1;Q3] | | |  |  |  |
| Total number of chronic conditions | | | 1.00 [0.00;2.00] | 0.00 [0.00;1.00] | *0.009* |
| Median [Q1;Q3] | | |  |  |  |
| Depression | Yes | | 8.1 | 6.2 | *0.112* |
|  | No | | 91.9 | 93.8 |  |
| Functioning and disability (WHODAS 2.0) | | | 9.44 [1.39;27.50] | 8.33 [0.83; 27.78] | *0.085* |
| Median [Q1;Q3] | | |  |  |  |
| Quality of life (WHOQOL-Age) | | | 65.89 (15.09) | 65.91 (15.22) | *0.980* |
| Mean (SD) | | |  |  |  |
| Loneliness | | | 0.00 [0.00;33.33] | 0.00 [0.00;33.33] | *0.684* |
| Median [Q1;Q3] | | |  |  |  |
| Using Internet | Yes | | 56.9 | 59.4 | *0.351* |
|  | No | | 43.1 | 40.6 |  |
| Frequency of using social media platforms (e.g., Facebook, Instagram, X) | Never | | 32.0 | 41.2 | *0.009* |
|  | Once or a few times a year | | 3.7 | 2.9 |  |
|  | Once or a few times a month | | 13.0 | 8.2 |  |
|  | Once or a few times a week | | 27.8 | 22.3 |  |
|  | Daily | | 23.5 | 25.5 |  |
| Frequency of using communication tools (e.g., WhatsApp, Messenger, Zoom, Skype) | Never | | 16.2 | 27.0 | *0.002* |
|  | Once or a few times a year | | 3.2 | 2.9 |  |
|  | Once or a few times a month | | 11.2 | 6.7 |  |
|  | Once or a few times a week | | 30.6 | 25.3 |  |
|  | Daily | | 38.8 | 38.1 |  |
| Frequency of sending/receiving emails in the last 12 months | Never | | 36.4 | 41.1 | *0.125* |
|  | Once or a few times a year | | 18.2 | 12.7 |  |
|  | Once or a few times a month | | 19.3 | 18.0 |  |
|  | Once or a few times a week | | 15.3 | 15.5 |  |
|  | Daily | | 10.8 | 12.8 |  |
| Frequency and type of online social contacts | Rare online contact | | 13.0 | 23.5 |  |
|  | Frequent online contact only through apps | | 20.6 | 19.2 | *0.001* |
|  | Frequent online contact through apps and social media platforms, monthly emails | | 59.2 | 50.7 |  |
|  | Frequent online contact through social media platforms, monthly emails, not apps | | 7.1 | 6.7 |  |

*Note:* ^1^ – unweighted number of respondents, *p value* for the Pearson chi-squared test with second-order Rao-Scott correction, Student’s t-test and Kruskal Wallis test as applicable

### Table S3.2a Frequencies of using SM platform, communication tools and emails in the last 12 months, unweighted data.

|  |  | **Women** | |  | **Men** | |  |
| --- | --- | --- | --- | --- | --- | --- | --- |
|  |  | Using the internet but not a member of online community (n=532) | Member of online community (n=83) |  | Using the internet but not a member of online community (n=387) | Member of online community (n=56) |  |
|  |  | n (%) | n (%) | *p value* | n (%) | n (%) | *p value* |
| Frequency of using social media platforms (e.g. Facebook, Instagram, X) | Never | 187 (35.2) | 3 (3.6) | *<0.001* | 182 (47.0) | 2 (3.6) | *<0.001* |
|  | Once or a few times a year | 18 (3.4) | 2 (2.4) |  | 9 (2.3) | 4 (7.1) |  |
|  | Once or a few times a month | 74 (13.9) | 10 (12.0) |  | 39 (10.1) | 2 (3.6) |  |
|  | Once or a few times a week | 144 (27.1) | 29 (34.9) |  | 83 (21.4) | 13 (23.2) |  |
|  | Daily | 109 (20.5) | 39 (47.0) |  | 74 (19.1) | 35 (62.5) |  |
| Frequency of using communication tools (e.g., WhatsApp, Messenger, Zoom, Skype | Never | 105 (19.7) | 1 (1.2) | *<0.001* | 111 (28.7) | 5 (8.9) | *<0.001 ^F^* |
|  | Once or a few times a year | 17 (3.2) | 3 (3.6) |  | 13 (3.4) | 1 (1.8) |  |
|  | Once or a few times a month | 61 (11.5) | 8 (9.6) |  | 29 (7.5) | 3 (5.4) |  |
|  | Once or a few times a week | 147 (27.6) | 24 (28.9) |  | 102 (26.4) | 12 (21.4) |  |
|  | Daily | 202 (38.0) | 47 (56.6) |  | 132 (34.1) | 35 (62.5) |  |
| Frequency of sending/receiving emails in the last 12 months | Never | 194 (36.5) | 17 (20.5) | *0.013* | 161 (41.6) | 13 (23.2) | *0.043* |
|  | Once or a few times a year | 87 (16.4) | 19 (22.9) |  | 44 (11.4) | 10 (17.9) |  |
|  | Once or a few times a month | 107 (20.1) | 17 (20.5) |  | 70 (18.1) | 11 (19.6) |  |
|  | Once or a few times a week | 82 (15.4) | 12 (14.5) |  | 63 (16.3) | 9 (16.1) |  |
|  | Daily | 62 (11.7) | 18 (21.7) |  | 49 (12.7) | 13 (23.2) |  |

| Table S3.3a Frequencies of using SM platform (e.g. Facebook, Instagram, X) for contacts with family, friends and acquaintances among women and men (n=1058), unweighted data. |
| --- |

|  |  | **Women** | **Men** | | | | |
| --- | --- | --- | --- | --- | --- | --- | --- |
|  |  | Using the internet but not a member of online community (n=532) | Member of online community (n=83) |  | Using the internet but not a member of online community (n=387) | Member of online community (n=56) |  |
|  |  | n (%) | n (%) | *p value* | n (%) | n (%) | *p value* |
| Frequency of social media contact with … **spouse or partner** | Never | 195 (70.9) | 30 (57.7) | *0.230* ^F^ | 96 (56.8) | 15 (40.5) | *0.215* ^F^ |
|  | Once or a few times a year | 16 (5.8) | 6 (11.5) |  | 9 (5.3) | 3 (8.1) |  |
|  | Once or a few times a month | 20 (7.3) | 5 (9.6) |  | 18 (10.7) | 8 (21.6) |  |
|  | Once or a few times a week | 30 (10.9) | 9 (17.3) |  | 30 (17.8) | 6 (16.2) |  |
|  | Daily | 14 (5.1) | 2 (3.8) |  | 16 (9.5) | 5 (13.5) |  |
| Frequency of social media contact with…**children** | Never | 135 (41.2) | 23 (32.4) | *0.415* | 84 (45.7) | 16 (35.6) | *0.637* ^F^ |
|  | Once or a few times a year | 37 (11.3) | 13 (18.3) |  | 19 (10.3) | 6 (13.3) |  |
|  | Once or a few times a month | 62 (18.9) | 15 (21.1) |  | 33 (17.9) | 11 (24.4) |  |
|  | Once or a few times a week | 76 (23.2) | 15 (21.1) |  | 39 (21.2) | 9 (20.0) |  |
|  | Daily | 18 (5.5) | 5 (7.0) |  | 9 (4.9) | 3 (6.7) |  |
| Frequency of social media contact with …**grandchildren** | Never | 117 (59.1) | 24 (58.5) | *0.045* ^F^ | 60 (57.1) | 15 (71.4) | *0.299* ^F^ |
|  | Once or a few times a year | 14 (7.1) | 8 (19.5) |  | 10 (9.5) | 4 (19.0) |  |
|  | Once or a few times a month | 39 (19.7) | 3 (7.3) |  | 17 (16.2) | 1 (4.8) |  |
|  | Once or a few times a week | 26 (13.1) | 5 (12.2) |  | 15 (14.3) | 1 (4.8) |  |
|  | Daily | 2 (1.0) | 1 (2.4) |  | 3 (2.9) | 0 (0.0) |  |
| Frequency of social media contact with …**other relatives** | Never | 108 (36.9) | 15 (23.8) | *0.048* ^F^ | 75 (44.9) | 12 (31.6) | *0.431* ^F^ |
|  | Once or a few times a year | 81 (27.6) | 21 (33.3) |  | 44 (26.3) | 13 (34.2) |  |
|  | Once or a few times a month | 93 (31.7) | 20 (31.7) |  | 39 (23.4) | 11 (28.9) |  |
|  | Once or a few times a week | 10 (3.4) | 6 (9.5) |  | 7 (4.2) | 1 (2.6) |  |
|  | Daily | 1 (0.3) | 1 (1.6) |  | 2 (1.2) | 1 (2.6) |  |
| Frequency of social media contact with …**friends** | Never | 58 (19.1) | 6 (8.2) | *<0.001* | 40 (23.7) | 5 (10.9) | *0.014* |
|  | Once or a few times a year | 78 (25.7) | 15 (20.5) |  | 36 (21.3) | 4 (8.7) |  |
|  | Once or a few times a month | 116 (38.2) | 29 (39.7) |  | 63 (37.3) | 20 (43.5) |  |
|  | Once or a few times a week | 48 (15.8) | 16 (21.9) |  | 20 (11.8) | 12 (26.1) |  |
|  | Daily | 4 (1.3) | 7 (9.6) |  | 10 (5.9) | 5 (10.9) |  |
| Frequency of social media contact with …**acquaintance** with whom meetings are infrequently | Never | 134 (38.8) | 15 (18.8) | <0.001 | 81 (39.5) | 8 (14.8) | <0.001 |
|  | Once or a few times a year | 119 (34.5) | 34 (42.5) |  | 65 (31.7) | 15 (27.8) |  |
|  | Once or a few times a month | 81 (23.5) | 20 (25.0) |  | 43 (21.0) | 20 (37.0) |  |
|  | Once or a few times a week | 11 (3.2) | 10 (12.5) |  | 15 (7.3) | 9 (16.7) |  |
|  | Daily | 0 (0.0) | 1 (1.3) |  | 1 (0.5) | 2 (3.7) |  |
| Frequency of social media contact with …**acquaintance** with whom no meetings | Never | 157 (45.5) | 16 (20.0) | *<0.001* ^F^ | 81 (39.5) | 8 (14.8) | *<0.001* ^F^ |
|  | Once or a few times a year | 117 (33.9) | 35 (43.8) |  | 66 (32.2) | 17 (31.5) |  |
|  | Once or a few times a month | 58 (16.8) | 21 (26.3) |  | 46 (22.4) | 21 (38.9) |  |
|  | Once or a few times a week | 13 (3.8) | 7 (8.8) |  | 10 (4.9) | 7 (13.0) |  |
|  | Daily | 0 (0.0) | 1 (1.3) |  | 2 (1.0) | 1 (1.9) |  |
| *Note: p value* for the Chi-squared test of independence (F - Fisher exact test) | | | | | | | |

### Table S3.3b Frequencies of using SM platform (e.g. Facebook, Instagram, X) for contacts with family, friends and acquaintances among women and men (n=1058), weighted data.

|  |  | **Women** | |  | **Men** | |  |
| --- | --- | --- | --- | --- | --- | --- | --- |
|  |  | Using the internet but not a member of online community (n^1^=532) | Member of online community (n=83) |  | Using the internet but not a member of online community (n=387) | Member of online community (n=56) |  |
|  |  | % | % | *p value* | % | % | *p value* |
| Frequency of social media contact with … **spouse or partner** | Never | 74.1 | 60.3 | *0.502* | 56.7 | 38.0 | *0.375* |
|  | Once or a few times a year | 5.1 | 8.4 |  | 5.4 | 9.2 |  |
|  | Once or a few times a month | 6.5 | 8.6 |  | 11.5 | 22.3 |  |
|  | Once or a few times a week | 9.9 | 16.7 |  | 16.6 | 19.8 |  |
|  | Daily | 4.4 | 5.9 |  | 9.8 | 10.7 |  |
| Frequency of social media contact with…**children** | Never | 43.2 | 36.1 | *0.703* | 44.5 | 33.6 | *0.647* |
|  | Once or a few times a year | 10.5 | 15.1 |  | 10.0 | 11.6 |  |
|  | Once or a few times a month | 18.8 | 19.3 |  | 16.9 | 26.8 |  |
|  | Once or a few times a week | 22.1 | 21.3 |  | 23.5 | 22.2 |  |
|  | Daily | 5.5 | 8.2 |  | 5.1 | 5.8 |  |
| Frequency of social media contact with …**grandchildren** | Never | 58.2 | 59.7 | *0.340^W^* | 58.9 | 67.1 | *0.257^W^* |
|  | Once or a few times a year | 6.7 | 16.5 |  | 9.9 | 22.7 |  |
|  | Once or a few times a month | 18.4 | 8.8 |  | 13.5 | 4.9 |  |
|  | Once or a few times a week | 15.3 | 12.7 |  | 14.7 | 5.2 |  |
|  | Daily | 1.4 | 2.3 |  | 3.0 | 0.0 |  |
| Frequency of social media contact with …**other relatives** | Never | 39.2 | 28.5 | *0.442^W^* | 44.0 | 31.4 | *0.708^W^* |
|  | Once or a few times a year | 27.1 | 28.4 |  | 25.0 | 36.0 |  |
|  | Once or a few times a month | 29.7 | 29.6 |  | 25.3 | 28.5 |  |
|  | Once or a few times a week | 3.9 | 12.0 |  | 4.1 | 3.0 |  |
|  | Daily | 0.1 | 1.5 |  | 1.5 | 1.2 |  |
| Frequency of social media contact with …**friends** | Never | 21.3 | 8.6 | *<0.001* | 23.7 | 13.9 | *0.110* |
|  | Once or a few times a year | 25.5 | 18.2 |  | 21.1 | 8.4 |  |
|  | Once or a few times a month | 38.4 | 41.6 |  | 35.2 | 45.8 |  |
|  | Once or a few times a week | 13.9 | 20.0 |  | 15.2 | 25.1 |  |
|  | Daily | 0.9 | 11.7 |  | 4.8 | 6.8 |  |
| Frequency of social media contact with …**acquaintance** with whom meetings are infrequently | Never | 41.2 | 19.4 | *0.006^W^* | 42.9 | 16.1 | *0.002^W^* |
|  | Once or a few times a year | 34.6 | 38.8 |  | 29.7 | 28.2 |  |
|  | Once or a few times a month | 21.3 | 26.7 |  | 20.2 | 37.5 |  |
|  | Once or a few times a week | 3.0 | 13.8 |  | 6.7 | 14.6 |  |
|  | Daily | 0.0 | 1.3 |  | 0.5 | 3.7 |  |
| Frequency of social media contact with …**acquaintance** with whom no meetings | Never | 49.3 | 19.6 | *<0.001^W^* | 42.5 | 17.4 | *0.007^W^* |
|  | Once or a few times a year | 31.7 | 43.8 |  | 30.5 | 32.1 |  |
|  | Once or a few times a month | 15.8 | 27.9 |  | 21.7 | 37.6 |  |
|  | Once or a few times a week | 3.2 | 7.3 |  | 4.3 | 12.0 |  |
|  | Daily | 0.0 | 1.3 |  | 1.0 | 0.8 |  |

*Note:* ^1^ – unweighted number of respondents; *p value* for the Pearson chi-squared test with second-order Rao-Scott correction, or when appropriate, the adjusted Wald test (W)

### Table S3.4a Frequencies of contact using communication tools (e.g., WhatsApp, Messenger, Zoom, Skype) with family, friends and acquaintances among women and men (n=1058), unweighted data.

|  |  | **Women** | |  | **Men** | |  |
| --- | --- | --- | --- | --- | --- | --- | --- |
|  |  | Using the internet but not a member of online community (n=532) | Member of online community (n=83) |  | Using the internet but not a member of online community (n=387) | Member of online community (n=56) |  |
|  |  | n (%) | n (%) | *p value* | n (%) | n (%) | *p value* |
| Frequency of contact using communication tools with … **spouse or partner** | Never | 185 (56.4) | 23 (42.6) | *0.325* ^F^ | 105 (46.7) | 8 (22.9) | *0.069* ^F^ |
|  | Once or a few times a year | 24 (7.3) | 4 (7.4) |  | 18 (8.0) | 3 (8.6) |  |
|  | Once or a few times a month | 27 (8.2) | 6 (11.1) |  | 27 (12.0) | 5 (14.3) |  |
|  | Once or a few times a week | 57 (17.4) | 12 (22.2) |  | 41 (18.2) | 10 (28.6) |  |
|  | Daily | 35 (10.7) | 9 (16.7) |  | 34 (15.1) | 9 (25.7) |  |
| Frequency of contact using communication tools with…**children** | Never | 117 (28.7) | 9 (12.2) | *0.008* ^F^ | 86 (34.3) | 7 (16.7) | *0.046* ^F^ |
|  | Once or a few times a year | 39 (9.6) | 6 (8.1) |  | 24 (9.6) | 4 (9.5) |  |
|  | Once or a few times a month | 69 (16.9) | 11 (14.9) |  | 43 (17.1) | 11 (26.2) |  |
|  | Once or a few times a week | 129 (31.6) | 31 (41.9) |  | 81 (32.3) | 13 (31.0) |  |
|  | Daily | 54 (13.2) | 17 (23.0) |  | 17 (6.8) | 7 (16.7) |  |
| Frequency of contact using communication tools with …**grandchildren** | Never | 109 (41.8) | 17 (39.5) | *0.311* ^F^ | 73 (48.3) | 7 (38.9) | *0.033* ^F^ |
|  | Once or a few times a year | 24 (9.2) | 5 (11.6) |  | 12 (7.9) | 6 (33.3) |  |
|  | Once or a few times a month | 52 (19.9) | 9 (20.9) |  | 32 (21.2) | 4 (22.2) |  |
|  | Once or a few times a week | 68 (26.1) | 8 (18.6) |  | 31 (20.5) | 1 (5.6) |  |
|  | Daily | 8 (3.1) | 4 (9.3) |  | 3 (2.0) | 0 (0.0) |  |
| Frequency of contact using communication tools with …**other relatives** | Never | 124 (34.5) | 10 (15.9) | *<0.001* ^F^ | 82 (36.6) | 9 (25.7) | *<0.001* |
|  | Once or a few times a year | 88 (24.5) | 12 (19.0) |  | 61 (27.2) | 8 (22.9) |  |
|  | Once or a few times a month | 113 (31.5) | 27 (42.9) |  | 55 (24.6) | 14 (40.0) |  |
|  | Once or a few times a week | 31 (8.6) | 12 (19.0) |  | 26 (11.6) | 2 (5.7) |  |
|  | Daily | 3 (0.8) | 2 (3.2) |  | 0 (0.0) | 2 (5.7) |  |
| Frequency of contact using communication tools with …**friends** | Never | 76 (20.5) | 5 (6.8) | *<0.001* ^F^ | 56 (24.2) | 3 (6.7) | *<0.001* ^F^ |
|  | Once or a few times a year | 64 (17.3) | 10 (13.5) |  | 52 (22.5) | 4 (8.9) |  |
|  | Once or a few times a month | 133 (35.8) | 21 (28.4) |  | 60 (26.0) | 15 (33.3) |  |
|  | Once or a few times a week | 91 (24.5) | 28 (37.8) |  | 59 (25.5) | 20 (44.4) |  |
|  | Daily | 7 (1.9) | 10 (13.5) |  | 4 (1.7) | 3 (6.7) |  |
| Frequency of contact using communication tools with …**acquaintance** with whom meetings are infrequently | Never | 180 (42.2) | 8 (9.8) | *<0.001* ^F^ | 108 (39.1) | 7 (13.7) | *<0.001* ^F^ |
|  | Once or a few times a year | 121 (28.3) | 27 (32.9) |  | 79 (28.6) | 17 (33.3) |  |
|  | Once or a few times a month | 108 (25.3) | 33 (40.2) |  | 72 (26.1) | 18 (35.3) |  |
|  | Once or a few times a week | 17 (4.0) | 12 (14.6) |  | 16 (5.8) | 8 (15.7) |  |
|  | Daily | 1 (0.2) | 2 (2.4) |  | 1 (0.4) | 1 (2.0) |  |
| Frequency of contact using communication tools with …**acquaintance** with whom no meetings | Never | 211 (49.4) | 13 (15.9) | *<0.001* ^F^ | 125 (45.3) | 7 (13.7) | *<0.001* ^F^ |
|  | Once or a few times a year | 122 (28.6) | 29 (35.4) |  | 76 (27.5) | 19 (37.3) |  |
|  | Once or a few times a month | 78 (18.3) | 31 (37.8) |  | 58 (21.0) | 19 (37.3) |  |
|  | Once or a few times a week | 15 (3.5) | 7 (8.5) |  | 16 (5.8) | 5 (9.8) |  |
|  | Daily | 1 (0.2) | 2 (2.4) |  | 1 (0.4) | 1 (2.0) |  |

*Note: p value* for the Chi-squared test of independence (F - Fisher exact test)

### Table S3.4b Frequencies of contact using communication tools (e.g., WhatsApp, Messenger, Zoom, Skype) with family, friends and acquaintances among women and men (n=1058), weighted data.

|  |  | **Women** | |  | **Men** | |  |
| --- | --- | --- | --- | --- | --- | --- | --- |
|  |  | Using the internet but not a member of online community (n^1^=532) | Member of online community (n=83) |  | Using the internet but not a member of online community (n=387) | Member of online community (n=56) |  |
|  |  | % | % | *p value* | % | % | *p value* |
| Frequency of contact using communication tools with … **spouse or partner** | Never | 61.0 | 41.0 | *0.080* | 46.6 | 22.2 | *0.090* |
|  | Once or a few times a year | 6.6 | 5.0 |  | 8.1 | 6.7 |  |
|  | Once or a few times a month | 7.8 | 13.9 |  | 12.0 | 15.2 |  |
|  | Once or a few times a week | 15.6 | 23.9 |  | 17.4 | 29.6 |  |
|  | Daily | 9.0 | 16.1 |  | 15.9 | 26.3 |  |
| Frequency of contact using communication tools with…**children** | Never | 29.9 | 13.5 | *0.026* | 33.2 | 16.2 | *0.080* |
|  | Once or a few times a year | 9.2 | 5.8 |  | 10.0 | 9.4 |  |
|  | Once or a few times a month | 17.2 | 16.9 |  | 16.2 | 25.2 |  |
|  | Once or a few times a week | 32.4 | 44.8 |  | 33.9 | 32.7 |  |
|  | Daily | 11.3 | 19.0 |  | 6.7 | 16.5 |  |
| Frequency of contact using communication tools with …**grandchildren** | Never | 41.2 | 39.4 | *0.599^W^* | 50.6 | 37.7 | *0.049^W^* |
|  | Once or a few times a year | 9.4 | 11.0 |  | 7.8 | 34.7 |  |
|  | Once or a few times a month | 21.3 | 21.9 |  | 19.3 | 22.7 |  |
|  | Once or a few times a week | 25.6 | 18.6 |  | 20.1 | 4.9 |  |
|  | Daily | 2.4 | 9.1 |  | 2.1 | 0.0 |  |
| Frequency of contact using communication tools with …**other relatives** | Never | 35.8 | 19.6 | *0.100^W^* | 35.6 | 28.8 | *0.063^W^* |
|  | Once or a few times a year | 24.8 | 18.4 |  | 29.2 | 21.5 |  |
|  | Once or a few times a month | 30.2 | 39.6 |  | 24.0 | 41.5 |  |
|  | Once or a few times a week | 7.9 | 20.6 |  | 11.2 | 4.1 |  |
|  | Daily | 1.4 | 1.8 |  | 0.0 | 4.1 |  |
| Frequency of contact using communication tools with …**friends** | Never | 22.1 | 7.7 | *<0.001* | 24.4 | 8.6 | *0.003^W^* |
|  | Once or a few times a year | 18.2 | 11.6 |  | 22.9 | 8.1 |  |
|  | Once or a few times a month | 35.2 | 28.9 |  | 25.4 | 35.4 |  |
|  | Once or a few times a week | 23.4 | 40.3 |  | 25.9 | 41.7 |  |
|  | Daily | 1.2 | 11.6 |  | 1.4 | 6.1 |  |
| Frequency of contact using communication tools with …**acquaintance** with whom meetings are infrequently | Never | 44.5 | 9.3 | *<0.001^W^* | 42.6 | 14.9 | *<0.001^W^* |
|  | Once or a few times a year | 28.4 | 34.2 |  | 27.3 | 34.8 |  |
|  | Once or a few times a month | 23.6 | 39.4 |  | 24.1 | 33.6 |  |
|  | Once or a few times a week | 3.4 | 15.2 |  | 5.7 | 14.8 |  |
|  | Daily | 0.1 | 1.9 |  | 0.3 | 1.9 |  |
| Frequency of contact using communication tools with …**acquaintance** with whom no meetings | Never | 52.7 | 14.4 | *<0.001^W^* | 49.0 | 15.1 | *<0.001^W^* |
|  | Once or a few times a year | 28.1 | 38.9 |  | 26.3 | 37.6 |  |
|  | Once or a few times a month | 16.1 | 36.6 |  | 19.2 | 35.6 |  |
|  | Once or a few times a week | 3.0 | 8.1 |  | 5.2 | 9.8 |  |
|  | Daily | 0.1 | 1.9 |  | 0.3 | 1.9 |  |

*Note:* ^1^ – unweighted number of respondents, *p value* for the Pearson chi-squared test with second-order Rao-Scott correction, or when appropriate, the adjusted Wald test (W)

### Table S3.5. Sociodemographic characteristics of participants by internet use and making online social connections across women (n=1038) and men (n=764), unweighted data.

|  |  |  |  | **Women** | | | |  | | **Men** | | | |  | |
| --- | --- | --- | --- | --- | --- | --- | --- | --- | --- | --- | --- | --- | --- | --- | --- |
|  |  |  |  | Not using the internet (n=423) | Using the internet but not a member of online community (n=532) | Member of online community (n=83) |  | | Not using the internet (n=321) | | Using the internet but not a member of online community (n=387) | Member of online community (n=56) |  | |  |
|  |  |  |  | n (%) | n (%) | n (%) | *p value* | | n (%) | | n (%) | n (%) | *p value* | |  |
| Age (years) Median [Q1;Q3] | | | | 70.00 [62.00;77.00] | 61.00 [54.00;67.00] | 57.00 [53.00;62.00] | *<0.001* | | 69.00 [62.00;76.00] | | 62.00 [54.00;67.00] | 58.00 [53.25;64.00] | *<0.001* | |  |
| Place of residence | Rural | | | 172 (40.7) | 193 (36.3) | 32 (38.6) | *0.715* | | 147 (45.8) | | 134 (34.6) | 22 (39.3) | *0.008* | |  |
|  | Urban – < 50 000 inhabitants | | | 102 (24.1) | 143 (26.9) | 21 (25.3) |  |  | 75 (23.4) | | 87 (22.5) | 15 (26.8) |  |  |  |
|  | Urban – 50 000 to 200 000 inh. | | | 80 (18.9) | 94 (17.7) | 13 (15.7) |  |  | 54 (16.8) | | 73 (18.9) | 12 (21.4) |  |  |  |
|  | Urban – > 200 000 inhabitants | | | 69 (16.3) | 102 (19.2) | 17 (20.5) |  |  | 45 (14.0) | | 93 (24.0) | 7 (12.5) |  |  |  |
| Marital status | Never been married | | | 17 (4.0) | 25 (4.7) | 6 (7.2) | *<0.001* | | 34 (10.6) | | 27 (7.0) | 9 (16.1) | *<0.001* | |  |
|  | Married | | | 190 (44.9) | 381 (71.6) | 49 (59.0) |  |  | 207 (64.5) | | 306 (79.1) | 37 (66.1) |  |  |  |
|  | Separated/Divorced | | | 32 (7.6) | 45 (8.5) | 16 (19.3) |  |  | 18 (5.6) | | 21 (5.4) | 7 (12.5) |  |  |  |
|  | Widowed | | | 184 (43.5) | 81 (15.2) | 12 (14.5) |  |  | 62 (19.3) | | 33 (8.5) | 3 (5.4) |  |  |  |
| Education level | Primary or lower | | | 125 (29.6) | 23 (4.3) | 1 (1.2) | *<0.001* | | 55 (17.1) | | 15 (3.9) | 1 (1.8) | *<0.001* | |  |
|  | Vocational | | | 169 (40.0) | 104 (19.5) | 19 (22.9) |  |  | 190 (59.2) | | 139 (35.9) | 21 (37.5) |  |  |  |
|  | Secondary | | | 111 (26.2) | 260 (48.9) | 30 (36.1) |  |  | 65 (20.2) | | 157 (40.6) | 14 (25.0) |  |  |  |
|  | Higher | | | 18 (4.3) | 145 (27.3) | 33 (39.8) |  |  | 11 (3.4) | | 76 (19.6) | 20 (35.7) |  |  |  |
| Individual monthly income in quintiles | ≤ 2 500 PLN | | | 195 (60.6) | 127 (30.4) | 18 (24.3) | *<0.001* | | 106 (39.6) | | 34 (11.7) | 6 (11.8) | *<0.001* | |  |
|  | 2 501 – 3 500 PLN | | | 70 (21.7) | 122 (29.2) | 15 (20.3) |  |  | 69 (25.7) | | 58 (19.9) | 8 (15.7) |  |  |  |
|  | 3 501 – 4 500 PLN | | | 23 (7.1) | 75 (17.9) | 13 (17.6) |  |  | 44 (16.4) | | 66 (22.7) | 15 (29.4) |  |  |  |
|  | 4 501 – 6 250 PLN | | | 13 (4.0) | 45 (10.8) | 13 (17.6) |  |  | 31 (11.6) | | 66 (22.7) | 8 (15.7) |  |  |  |
|  | > 6 250 PLN | | | 21 (6.5) | 49 (11.7) | 15 (20.3) |  |  | 18 (6.7) | | 67 (23.0) | 14 (27.5) |  |  |  |
| Subjective SES Mean (SD) | | | | 5.34 (1.56) | 6.42 (1.57) | 6.55 (1.78) | *<0.001* | | 5.66 (1.59) | | 6.50 (1.50) | 6.69 (1.71) | *<0.001* | |  |

*Note:* SD - Standard Deviation; Q1 - 25th Percentile; Q3 - 75th Percentile; p value for the Chi-squared test of independence; one-way ANOVA or Kruskal-Wallis test as applicable

### Table S3.6. Health and functional status characteristics, social networks, loneliness and QoL of participants by internet use and making online social connections across women (n=1038) and men (n=764), unweighted data.

|  |  |  |  | **Women** | | |  | **Men** | | |  |
| --- | --- | --- | --- | --- | --- | --- | --- | --- | --- | --- | --- |
|  |  |  |  | Not using the internet (n=423) | Using the internet but not a member of online community (n=532) | Member of online community (n=83) | *p value* | Not using the internet (n=321) | Using the internet but not a member of online community (n=387) | Member of online community (n=56) | *p value* |
| Total number of chronic conditions | | | | 1.00 [0.00;2.00] | 0.00 [0.00;1.00] | 1.00 [0.00;1.00] | *<0.001* | 1.00 | 0.00 [0.00;1.00] | 0.00 [0.00;1.00] | *0.002* |
| Median [Q1;Q3] | | | |  |  |  |  | [0.00;2.00] |  |  |  |
| Depression [n(%)] | Yes | | | 46 (10.9) | 29 (5.5) | 8 (9.6) | *0.008* | 27 (8.4) | 17 (4.4) | 3 (5.4) | *0.083* |
|  | No | | | 377 (89.1) | 503 (94.5) | 75 (90.4) |  | 294 (91.6) | 370 (95.6) | 53 (94.6) |  |
| Functioning and disability (WHODAS 2.0) Median [Q1;Q3] | | | | 22.22 [7.78;42.22] | 4.17 [0.56;12.71] | 5.56 [1.39;15.00] | *<0.001* | 16.67 [3.19; 38.26] | 3.61 [0.56;16.39] | 2.92 [0.56;15.63] | *<0.001* |
| Level of social networks (COURAGE – SNI)  Median [Q1;Q3] | | | | 57.48 [48.89;65.32] | 60.87 [54.00;68.62] | 67.62 [58.14;75.33] | *<0.001* | 58.89 [50.73;66.02] | 61.66 [52.85;68.43] | 62.77 [52.54;69.01] | *0.005* |
| Quality of life (WHOQOL-Age) | | | | 61.45 [50.00;69.37] | 72.09 [64.29;77.92] | 73.81 [67.86;80.49] | *<0.001* | 62.09 | 71.89 [64.24;77.75] | 74.13 [64.81;79.76] | *<0.001* |
| Median [Q1;Q3] | | | |  |  |  |  | [50.98;70.99] |  |  |  |
| Loneliness  Median [Q1;Q3] | | | | 11.11 [0.00;33.33] | 0.00 [0.00;22.22] | 11.11 [0.00;33.33] | *<0.001* | 11.11 [0.00;33.33] | 0.00 [0.00;22.22] | 0.00 [0.00;22.22] | *0.230* |

*Note:* Q1 - 25th Percentile; Q3 - 75th Percentile; *p value* for the Chi-squared test of independence or Kruskal-Wallis test as applicab

### Table S3.7. Online social contacts characteristics of participants by making online social connections across women (n=615) and men (n=443), unweighted data.

|  |  | **Women** | | | **Men** | | | | | |
| --- | --- | --- | --- | --- | --- | --- | --- | --- | --- | --- |
|  |  | Using the internet but not a member of online community (n=532) | Member of online community (n=83) |  | Using the internet but not a member of online community (n=387) | Member of online community (n=56) |  | | | |
|  |  | n (%) | n (%) | *p value* | n (%) | n (%) | *p value* | | | |
| Frequency and type of online social contacts | Rare online contact | 84 (15.8) | 2 (2.4) |  | 100 (25.8) | 1 (1.8) |  | | | |
|  | Frequent online contact only through apps | 111 (20.9) | 2 (2.4) | *<0.001* | 85 (22.0) | 4 (7.1) | *<0.001* |  |  |  |
|  | Frequent online contact through apps and social media platforms, monthly emails | 295 (44.7) | 76 (91.6) |  | 177 (45.7) | 46 (82.1) |  |  |  |  |
|  | Frequent online contact through social media platforms, monthly emails, not apps | 42 (7.9) | 3 (3.6) |  | 25(6.5) | 5 (8.9) |  |  |  |  |
| Number of Internet contacts per week | Lack of such people | 172 (35.6) | 0 (0.0) | *<0.001* | 136 (41.8) | 0 (0.0) | *<0.001* ^F^ |  |  |  |
|  | 1 person | 15 (3.1) | 2 (2.4) |  | 5 (1.5) | 0 (0.0) |  |  |  |  |
|  | 2 to 3 people | 94 (19.5) | 11 (13.3) |  | 47 (14.5) | 6 (10.7) |  |  |  |  |
|  | 4 to 5 people | 73 (15.1) | 15 (18.1) |  | 53 (16.3) | 10 (17.9) |  |  |  |  |
|  | 6 to 10 people | 85 (17.6) | 22 (26.5) |  | 44 (13.5) | 8 (14.3) |  |  |  |  |
|  | 11 to 20 people | 31 (6.4) | 19 (22.9) |  | 25 (7.7) | 13 (23.2) |  |  |  |  |
|  | 21 people or more | 13 (2.7) | 14 (16.9) |  | 15 (4.6) | 19 (33.9) |  |  |  |  |
| Number of people contacted online and then face-to-face | Lack of such people | 272 (87.5) | 46 (55.4) | *<0.001* ^F^ | 153 (81.0) | 21 (37.5) | *<0.001* ^F^ |  |  |  |
|  | 1 person | 14 (4.5) | 9 (10.8) |  | 9 (4.8) | 9 (16.1) |  |  |  |  |
|  | 2 to 3 people | 19 (6.1) | 15 (18.1) |  | 19 (10.1) | 11 (19.6) |  |  |  |  |
|  | 4 to 5 people | 3 (1.0) | 6 (7.2) |  | 3 (1.6) | 2 (3.6) |  |  |  |  |
|  | 6 people or more | 3 (1.0) | 7 (8.4) |  | 5 (2.6) | 13 (23.2) |  |  |  |  |
| Number of people contacted online but not known in person, during the last 12 months | Lack of such people | 237 (76.2) | 28 (33.7) | *<0.001* ^F^ | 122 (64.6) | 8 (14.3) | *<0.001* ^F^ |  |  |  |
|  | 1 person | 10 (3.2) | 5 (6.0) |  | 6 (3.2) | 3 (5.4) |  |  |  |  |
|  | 2 to 3 people | 31 (10.0) | 19 (22.9) |  | 29 (15.3) | 11 (19.6) |  |  |  |  |
|  | 4 to 5 people | 17 (5.5) | 11 (13.3) |  | 14 (7.4) | 7 (12.5) |  |  |  |  |
|  | 6 to 10 people | 12 (3.9) | 10 (12.0) |  | 7 (3.7) | 7 (12.5) |  |  |  |  |
|  | 11 to 20 people | 2 (0.6) | 5 (6.0) |  | 3 (1.6) | 5 (8.9) |  |  |  |  |
|  | 21 people or more | 2 (0.6) | 5 (6.0) |  | 8 (4.2) | 15 (26.8) |  |  |  |  |
| *Note:* *p value* for the Chi-squared test of independence (F- Fisher exact test) | | | | | | | | | |  |

## Cluster analysis

### Table S3.8. Cluster means for social media, communication apps, and email use frequencies for each cluster solution.

|  | Cluster | | | |
| --- | --- | --- | --- | --- |
|  | 1  (Rare online contact)  **n=187** | 2  (Frequent online contact through apps, occasional emails, not social media platforms)  **n=202** | 3  (Frequent online contact through apps and social media platforms, monthly emails)  **n=594** | 4  (Frequent online contact through social media platforms, monthly emails, not apps)  **n=75** |
| Frequency of using communication tools (e.g., WhatsApp, Messenger, Zoom, Skype) | 1.05 | 4.23 | 4.46 | 1.47 |
| Frequency of using social media platforms (e.g., Facebook, Instagram, X) | 1.02 | 1.06 | 2.85 | 2.93 |
| Frequency of sending/receiving emails | 1.91 | 2.11 | 4.20 | 3.69 |
|  |  |  |  |  |
| Clusterwise Jaccard bootstrapmean: | 0.9497 | 0.9613 | 0.9546 | 0.8233 |

*Note:* We evaluated multiple clustering approaches, such as k-means and hierarchical clustering (hclust), testing various configurations including Euclidean, Manhattan, and Gower distances, as well as average and Ward linkages. The most stable results were obtained using hierarchical clustering with the Euclidean distance metric and the average linkage method.

The selection of the number of clusters was based on the NbClust method (R software; distance measure: Euclidean; clustering method: average). Among all indices, 6 proposed 2 as the optimal number of clusters, while 5 proposed 4. Although 2 was the most frequently suggested option, 3 clusters were ultimately chosen because this solution provided greater differentiation among respondents and allowed for more meaningful interpretation. Clusterwise Jaccard bootstrap mean was used to assess stability the results. The cut-off value which confirm stability of the results is 0.80 (ref: Yu, H., Chapman, B., Di Florio, A., Eischen, E., Gotz, D., Jacob, M., & Blair, R. H. (2019). Bootstrapping estimates of stability for clusters, observations and model selection. Computational Statistics, 34(1), 349-372.)

## Regression models

### Table S3.9. Simple Slopes Analysis: Conditional Effects of Loneliness on Quality of Life Across Internet Use and Making Online Social Connections for Women. Weighted data.

|  | Not using the Internet | | | Member of online community | | | Using the Internet but not a member of online community | | |
| --- | --- | --- | --- | --- | --- | --- | --- | --- | --- |
| Loneliness Effect | β | 95% CI | *p value* | β | 95% CI | *p value* | β | 95% CI | *p value* |
| Model 1 | -0.31 | (-0.37,-0.26) | *<0.001* | -0.08 | (-0.18,0.01) | *0.091* | -0.30 | (-0.38,-0.23) | *<0.001* |
| Model 2 | -0.29 | (-0.35,-0.24) | *<0.001* | -0.07 | (-0.16,0.024) | *0.145* | -0.26 | (-0.35,-0.16) | *<0.001* |
| Model 3 | -0.20 | (-0.26,-0.13) | *<0.001* | -0.06 | (-0.16,0.04) | *0.238* | -0.21 | (-0.30,-0.12) | *<0.001* |
| Model 4 | -0.16 | (-0.22,-0.10) | *<0.001* | -0.05 | (-0.15,0.05) | *0.323* | -0.19 | (-0.28,-0.09) | *<0.001* |

*Note: β -* unstandardized regression coefficients; 95% CI - 95% Confidence Interval; Model 1 - adjusted for age; Model 2 - additionally adjusted for both objective and subjective SES, place of residence and marital status; Model 3 - additionally adjusted for health (presence of depression, total number of chronic conditions, functioning and disability); Model 4 - additionally adjusted for Social Network Index

### Table S3.10. Simple Slopes Analysis: Conditional Effects of Loneliness on Quality of Life Across Frequency and Type of Online Social Contacts for women (model 4). Weighted data.

|  | Rare online contact | | | Frequent online contact only through apps | | | Frequent online contact through apps and SM platforms, monthly emails | | | Frequent online contact through SM plaforms, monthly emails, not apps | | |
| --- | --- | --- | --- | --- | --- | --- | --- | --- | --- | --- | --- | --- |
| Loneliness Effect | β | 95% CI | *p value* | β | 95% CI | *p value* | β | 95% CI | *p value* | β | 95% CI | *p value* |
| Model 1 | -0.41 | (-0.57,-0.25) | *<0.001* | -0.26 | (-0.38,-0.13) | *<0.001* | -0.22 | (-0.31,-0.13) | *<0.001* | -0.23 | (-0.4,-0.06) | *0.008* |
| Model 2 | -0.41 | (-0.65,-0.17) | *<0.001* | -0.15 | (-0.26,-0.04) | *0.007* | -0.21 | (-0.3,-0.12) | *<0.001* | -0.22 | (-0.4,-0.04) | *0.017* |
| Model 3 | -0.38 | (-0.63,-0.12) | *0.004* | -0.08 | (-0.18,0.03) | *0.139* | -0.17 | (-0.24,-0.09) | *<0.001* | -0.19 | (-0.31,-0.08) | *0.002* |
| Model 4 | -0.35 | (-0.63,-0.07) | *0.016* | -0.06 | (-0.17,0.04) | *0.248* | -0.16 | (-0.23,-0.09) | *<0.001* | -0.18 | (-0.3,-0.05) | *0.005* |

*Note: β -* unstandardized regression coefficients; 95% CI - 95% Confidence Interval; Model 1 - adjusted for age; Model 2 - additionally adjusted for both objective and subjective SES, place of residence and marital status; Model 3 - additionally adjusted for health (presence of depression, total number of chronic conditions, functioning and disability); Model 4 - additionally adjusted for Social Network Index

### Table S3.11. Linear regression models: associations between loneliness and QoL, without interactions. Unweighted data.

| Dependent variable: Quality of Life | **Women** | | | | | | | |
| --- | --- | --- | --- | --- | --- | --- | --- | --- |
|  | Model 1 | | Model 2 | | Model 3 | | Model 4 | |
|  | β (SE) | *p value* | β (SE) | *p value* | β (SE) | *p value* | β (SE) | *p value* |
| Loneliness | -0.32 (0.02) | *<0.001* | -0.27 (0.02) | *<0.001* | -0.19 (0.02) | *<0.001* | -0.15 (0.02) | *<0.001* |
| Dependent variable: Quality of Life | **Men** | | | | | | | |
|  | Model 1 | | Model 2 | | Model 3 | | Model 4 | |
|  | β (SE) | *p value* | β (SE) | *p value* | β (SE) | *p value* | β (SE) | *p value* |
| Loneliness | -0.33 (0.02) | *<0.001* | -0.25 (0.02) | *<0.001* | -0.17 (0.02) | *<0.001* | -0.15 (0.02) | *<0.001* |
| *Note:* SE - standard error; Model 1 - adjusted for age; Model 2 - additionally adjusted for both objective and subjective SES, place of residence and marital status; Model 3 - additionally adjusted for health (presence of depression, total number of chronic conditions, functioning and disability); Model 4 - additionally adjusted for Social Network Index | | | | | | | | |

### Table S3.12. Linear regression models: associations between loneliness and QoL, without interactions. Weighted data.

| Dependent variable: Quality of Life | **Women** | | | | | | | |
| --- | --- | --- | --- | --- | --- | --- | --- | --- |
|  | Model 1 | | Model 2 | | Model 3 | | Model 4 | |
|  | β (SE) | *p value* | β (SE) | *p value* | β (SE) | *p value* | β (SE) | *p value* |
| Loneliness | -0.31 (0.02) | *<0.001* | -0.26 (0.02) | *<0.001* | -0.19 (0.03) | *<0.001* | -0.16 (0.03) | *<0.001* |
| Dependent variable: Quality of Life | **Men** | | | | | | | |
|  | Model 1 | | Model 2 | | Model 3 | | Model 4 | |
|  | β (SE) | *p value* | β (SE) | *p value* | β (SE) | *p value* | β (SE) | *p value* |
| Loneliness | -0.32 (0.03) | *<0.001* | -0.24 (0.03) | *<0.001* | -0.15 (0.03) | *<0.001* | -0.15 (0.02) | *<0.001* |
| *Note:* SE - standard error; Model 1 - adjusted for age; Model 2 - additionally adjusted for both objective and subjective SES, place of residence and marital status; Model 3 - additionally adjusted for health (presence of depression, total number of chronic conditions, functioning and disability); Model 4 - additionally adjusted for Social Network Index | | | | | | | | |
