# Supplementary material for: Personal online social networks as moderators of the association between loneliness and quality of life in Polish adults aged 50+
Source: Sci Rep. 2025 Nov 19;15:40797. doi: 10.1038/s41598-025-24545-z (PMC12630643; doi:10.1038/s41598-025-24545-z)
Supplement: Supplementary file 4 — Supplementary Material 4 [file 41598_2025_24545_MOESM4_ESM.docx]

**Personal online social networks as moderators of the association between loneliness and quality of life in Polish adults aged 50+**

Supplementary file 4

Table of contents

[Table S4.1. Linear regression models examining the moderating role of ‘internet use and making online social connections’ after the imputation of objective and subjective SES, weighted data. 3](#_Toc208575147)

[Table S4.2. Linear regression models examining the moderating role of ‘frequency and type of online social contacts’ after the imputation of objective and subjective SES, weighted data. 4](#_Toc208575148)

**Statistical analysis**

In the main analysis, listwise deletion was applied to cases with missing data in income and subjective economic status. To assess whether the data were missing completely at random (MCAR), Little’s test (Little, 1988) was conducted using the *naniar* package in R. The results indicated that the null hypothesis of MCAR was rejected (χ² = 39.5, df = 22, p = 0.0123; number of missing data patterns = 3).

Therefore, an additional analysis was performed using multiple imputation in SPSS (method: regression models). Ten imputed datasets were subsequently analyzed in R using the *survey, mice,* and *mitools* packages. The pooled results of the regression models are presented in tables S4.1 and S4.2.

### Table S4.1. Linear regression models examining the moderating role of ‘internet use and making online social connections’ after the imputation of objective and subjective SES, weighted data.

| Dependent variable: Quality of Life | | **Women** | | | | | | | | | |
| --- | --- | --- | --- | --- | --- | --- | --- | --- | --- | --- | --- |
|  |  | **Model 1** | | **Model 2** | | **Model 3** | | | **Model 4** | | |
|  |  | β (SE) | *p value* | β (SE) | *p value* | | β (SE) | *p value* | | β (SE) | *p value* |
| **Loneliness** | | -0.32 (0.03) | *<0.001* | -0.29 (0.03) | *<0.001* | | -0.20 (0.03) | *<0.001* | | -0.16 (0.03) | *<0.001* |
| **Internet use and making online social connections** (Ref. Not using the internet) | | | | | | | | | | | |
|  | Using the internet but not a member of online community | 7.71 (1.13) | *<0.001* | 4.23 (1.20) | *0.001* | | 2.82 (1.16) | *0.016* | | 2.77 (1.07) | *0.011* |
|  | Member of online community | 7.27 (1.96) | *<0.001* | 3.05 (1.88) | *0.106* | | 3.18 (1.81) | *0.081* | | 2.52 (1.84) | *0.172* |
| **Loneliness * Internet use and making online social connections** | | | | | | | | | | | |
|  | Using the internet but not a member of online community | 0.01 (0.04) | *0.799* | 0.03 (0.05) | *0.566* | | -0.01 (0.05) | *0.827* | | -0.02 (0.04) | *0.727* |
|  | Member of online community | 0.23 (0.06) | *<0.001* | 0.24 (0.05) | *<0.001* | | 0.15 (0.05) | *0.004* | | 0.12 (0.05) | *0.027* |
| Dependent variable: Quality of Life | | **Men** | | | | | | | | | |
|  |  | **Model 1** | | **Model 2** | | **Model 3** | | | **Model 4** | | |
|  |  | β (SE) | *p value* | β (SE) | *p value* | | β (SE) | *p value* | | β (SE) | *p value* |
| **Loneliness** | | -0.32 (0.04) | *<0.001* | -0.28 (0.04) | *<0.001* | | -0.17 (0.04) | *<0.001* | | -0.16 (0.04) | *<0.001* |
| **Internet use and making online social connections** (Ref. Not using the internet) | | | | | | | | | | | |
|  | Using the internet but not a member of online community | 8.33 (1.42) | *<0.001* | 3.98 (1.43) | *0.006* | | 2.77 (1.32) | *0.038* | | 2.99 (1.28) | *0.021* |
|  | Member of online community | 12.63 (2.31) | *<0.001* | 6.81 (2.47) | *0.007* | | 5.55 (2.14) | *0.011* | | 5.38 (2.13) | *0.013* |
| **Loneliness * Internet use and making online social connections** | | | | | | | | | | | |
|  | Using the internet but not a member of online community | 0.02 (0.05) | *0.743* | 0.04 (0.05) | *0.477* | | 0.01 (0.05) | *0.767* | | 0.01 (0.05) | *0.872* |
|  | Member of online community | -0.07 (0.11) | *0.554* | 0.00 (0.10) | *0.998* | | -0.03 (0.09) | *0.715* | | -0.03 (0.08) | *0.728* |

### Table S4.2. Linear regression models examining the moderating role of ‘frequency and type of online social contacts’ after the imputation of objective and subjective SES, weighted data.

| Dependent variable: Quality of Life | | **Women** | | | | | | | |
| --- | --- | --- | --- | --- | --- | --- | --- | --- | --- |
|  |  | **Model 1** | | **Model 2** | | **Model 3** | | **Model 4** | |
|  |  | β (SE) | *p value* | β (SE) | *p value* | β (SE) | *p value* | β (SE) | *p value* |
| **Loneliness** | | -0.41 (0.08) | *<0.001* | -0.38 (0.10) | *<0.001* | -0.34 (0.11) | *0.003* | -0.29 (0.12) | *0.019* |
| **Frequency and type of online social contacts** (Ref. Rare online contact) | | | | | | | | | |
|  | Frequent online contact only through apps | -1.76 (2.38) | *0.461* | -2.69 (2.38) | *0.261* | -2.96 (2.23) | *0.188* | -3.49 (2.12) | *0.103* |
|  | Frequent online contact through apps and social media platforms, monthly emails | -1.55 (2.19) | *0.481* | -2.61 (2.2) | *0.238* | -1.83 (2.03) | *0.371* | -2.55 (1.87) | *0.176* |
|  | Frequent online contact through social media platforms, monthly emails, not apps | 3.21 (2.85) | *0.262* | 1.92 (2.66) | *0.472* | 2.52 (2.52) | *0.321* | 2.08 (2.54) | *0.415* |
| **Loneliness * Frequency and type of online social contacts** (Ref. Rare online contact) | | | | | | | | | |
|  | Frequent online contact only through apps | 0.15 (0.10) | *0.156* | 0.18 (0.12) | *0.138* | 0.24 (0.11) | *0.037* | 0.21 (0.12) | *0.076* |
|  | Frequent online contact through apps and social media platforms, monthly emails | 0.18 (0.10) | *0.074* | 0.18 (0.12) | *0.120* | 0.19 (0.11) | *0.103* | 0.15 (0.12) | *0.218* |
|  | Frequent online contact through social media platforms, monthly emails, not apps | 0.18 (0.12) | *0.140* | 0.18 (0.13) | *0.161* | 0.15 (0.11) | *0.190* | 0.13 (0.12) | *0.298* |
| Dependent variable: Quality of Life | | **Men** | | | | | | | |
|  |  | **Model 1** | | **Model 2** | | **Model 3** | | **Model 4** | |
|  |  | β (SE) | *p value* | β (SE) | *p value* | β (SE) | *p value* | β (SE) | *p value* |
| **Loneliness** | | -0.37 (0.06) | *<0.001* | -0.35 (0.06) | *<0.001* | -0.25 (0.05) | *<0.001* | -0.24 (0.05) | *<0.001* |
| **Frequency and type of online social contacts** (Ref. Rare online contact) | | | | | | | | | |
|  | Frequent online contact only through apps | 0.68 (2.11) | *0.749* | 0.34 (2.04) | *0.868* | 0.23 (1.67) | *0.892* | 0.00 (1.62) | *0.998* |
|  | Frequent online contact through apps and social media platforms, monthly emails | 0.92 (1.82) | *0.616* | -1.14 (1.97) | *0.566* | -1.12 (1.57) | *0.480* | -1.35 (1.54) | *0.381* |
|  | Frequent online contact through social media platforms, monthly emails, not apps | 0.25 (2.64) | *0.925* | 2.01 (2.64) | *0.449* | 0.63 (2.27) | *0.783* | 0.48 (2.19) | *0.827* |
| **Loneliness * Frequency and type of online social contacts** | | | | | | | | | |
|  | Frequent online contact only through apps | 0.12 (0.09) | 0.152 | 0.12 (0.08) | *0.134* | 0.10 (0.08) | *0.225* | 0.11 (0.08) | *0.214* |
|  | Frequent online contact through apps and social media platforms, monthly emails | 0.07 (0.07) | 0.312 | 0.12 (0.07) | *0.085* | 0.10 (0.06) | *0.094* | 0.10 (0.06) | *0.079* |
|  | Frequent online contact through social media platforms, monthly emails, not apps | 0.16 (0.11) | 0.156 | 0.16 (0.09) | *0.087* | 0.13 (0.08) | *0.094* | 0.15 (0.08) | *0.060* |

*Note*: models examining the moderator’s role in the association between loneliness and quality of life; β – unstandardized regression coefficient, SE - standard error; Model 1 - adjusted for age; Model 2 - additionally adjusted for both objective and subjective SES, place of residence and  marital status ; Model 3 - additionally adjusted for health (presence of depression, total number of chronic conditions, functioning and disability); Model 4 - additionally adjusted for level of social network
